# Bioactive Silk Sericin/Bioceramic Nerve Guidance Conduit for Effective Repair of Long‐Gap Transected Peripheral Nerve Injury through Regulating Schwann Cells

**DOI:** 10.1002/advs.202507241

**Published:** 2025-07-08

**Authors:** Qiangfei Su, Jian Wang, Yu Song, Zhaowenbin Zhang, Bo Cai, Xiakeerzhati Xiaohalati, Jingwei Liu, Haozhe Li, Zheng Wang, Jiang Chang, Lin Wang

**Affiliations:** ^1^ Department of Clinical Laboratory Union Hospital Tongji Medical College Huazhong University of Science and Technology Wuhan 430022 China; ^2^ Hubei Key Laboratory of Regenerative Medicine and Multi‐disciplinary Translational Research Hubei Provincial Engineering Research Center of Clinical Laboratory and Active Health Smart Equipment Research Center for Tissue Engineering and Regenerative Medicine Union Hospital Tongji Medical College Huazhong University of Science and Technology Wuhan 430022 China; ^3^ Center for Science & Technology Development Shenzhen Huazhong University of Science and Technology Research Institute Shenzhen 518057 China; ^4^ State Key Laboratory of High Performance Ceramics and Superfine Microstructure Shanghai Institute of Ceramics Chinese Academy of Sciences Shanghai 200050 China; ^5^ Zhejiang Engineering Research Center for Tissue Repair Materials Wenzhou Institute University of Chinese Academy of Sciences Wenzhou 325000 China; ^6^ Department of Gastrointestinal Surgery Union Hospital Tongji Medical College Huazhong University of Science and Technology Wuhan 430022 China

**Keywords:** akermanite, ion‐based therapy, long‐gap nerve regeneration, nerve guidance conduit, Schwann cell function, silk sericin

## Abstract

Schwann cells are pivotal in generating a pro‐regenerative microenvironment for long‐gap peripheral nerve injury (PNI) repair via their orchestrated behaviors, including cell migration, proliferation, and secretion. Bioceramics can release bioactive ions to regulate “repair” cells for regenerating damaged tissues. Herein, bioceramic akermanite (AT) is screened and found to significantly enhance Schwann cell proliferation, migration, and secretion by activating the PI3K/AKT and MAPK/ERK signaling pathways. Integration with silk sericin (SS), a natural biomaterial possessing excellent bioactivity, promotes the release of Ca and Mg from AT, synergistically enhancing Schwann cell pro‐regenerative behaviors and accelerating axon elongation. The AT‐SS composite conduit effectively restores nerve structure and function in a 13 mm transected PNI. Compared with commercial eton conduit, AT‐SS conduit promotes axons and myelin sheaths regeneration, improves nerve conduction, and effectively alleviates gastrocnemius muscle atrophy. The AT‐SS conduit achieves autograft‐comparable behavioral recovery as evidenced by the paw withdrawal latency, hind limb grip force, and sciatic function index. The excellent degradation and biosafety of AT‐SS conduit indicate its potential for clinical translation. This study introduces an ion‐based therapeutic approach for enhancing the pro‐regenerative functions of Schwann cells, and provides novel insights and strategies for clinically managing long‐gap PNI and other nerve tissue injuries.

## Graphical abstract

This study identifies akermanite among six bioceramics and reveals its ability to enhance Schwann cell's proliferation, migration, and secretion. When combined with silk sericin, the composite conduit synergistically enhances Schwann cell‐mediated regenerative processes. In 13 mmlong‐gap sciatic nerve defect repair, it achieves comparable recovery to autografts while outperforming commercial alternative.

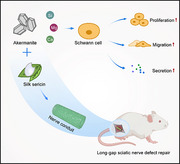

## 1 Introduction

Peripheral nerve injury (PNI) causes sensory loss and motor dysfunction, typically resulting in severe disabilities and poor outcomes, seriously decreasing the life quality of patients.^[^ ^1^, ^2^ ^]^ Clinically, there are three common approaches for treating such injuries. End‐to‐end neuroanastomosis is primarily used to repair short‐gap PNI. Autologous nerve grafting is regarded as the “gold standard” for treating long‐gap peripheral nerve defects (>10 mm).^[^ ^3^ ^]^ However, it is limited by mismatches between donor and recipient nerves, secondary injuries to donor sites, and the risk of neuroma formation.^[^ ^4^ ^]^ Currently, artificial nerve guidance conduits (NGCs), which circumvent the aforementioned limitations, have been developed to bridge nerve stumps and are regarded as promising substitutes for autologous nerve grafting.^[^ ^5^ ^]^ Although several NGCs, such as Neurotube, Neurolac, and NeuraGen, have been approved for clinical use, they are restricted to treating short‐distance nerve defects.^[^ ^6^, ^7^, ^8^, ^9^ ^]^ For long‐gap PNI with limiting sizes of 13 mm or more, these existing NGCs are insufficient and result in unsatisfactory functional recovery.^[^ ^10^, ^11^ ^]^

Schwann cells, principal glial cells of the peripheral nervous system, play a crucial role in nerve regeneration. Following long‐gap PNI, Schwann cells proliferate and migrate to form Büngner bands, guiding axonal regrowth, while secreting diverse factors to establish a permissive microenvironment for nerve regeneration.^[^ ^12^, ^13^ ^]^ Supplementation with allogeneic Schwann cells is a common approach for enhancing long‐gap PNI regeneration.^[^ ^14^, ^15^ ^]^ However, it is limited by low cell survival rates after in vivo implantation. Additionally, topological microstructures, such as chiral fibers, multichannel, and aligned microchannel structures, have been constructed to promote the secretion and migration of Schwann cells during the preparation of NGCs,^[^ ^16^, ^17^, ^18^, ^19^, ^20^ ^]^ however, poor degradability and unsatisfactory functional recovery limit their clinical translation. Furthermore, incorporating neurotrophic factors into NGCs is a straightforward approach to promote nerve regeneration. However, the unstable bioactivity and short half‐life of neurotrophic factors restrict their applications.^[^ ^21^, ^22^ ^]^ Moreover, electrical stimulation has been shown to boost the Schwann cells secretion of neurotrophic factors, thereby promoting long‐gap peripheral nerve regeneration.^[^ ^23^, ^24^ ^]^ Nevertheless, the preparation of a highly compatible and implantable electrical device with stable powering performance remains challenging. To our knowledge, none of the available NGCs can simultaneously activate Schwann cell proliferation, migration, and cytokine secretion, all critical for long‐gap nerve regeneration.

Bioactive ions are ionic species (e.g., Ca, Mg, Sr, and Si ions) capable of regulating cellular biological functions and influencing multiple physiological/pathological processes.^[^ ^25^, ^26^, ^27^, ^28^ ^]^ Specific bioactive ions have been shown to regulate regeneration‐promoting cell behaviors, including cell proliferation, migration, and cytokine secretion. For example, Si ions enhance VEGF expression and osteoblast‐like cell proliferation and promote angiogenesis and bone regeneration; Ca ions increase Schwann cell migration and accelerate axon outgrowth;^[^ ^29^, ^30^, ^31^, ^32^ ^]^ and Mg ions can enhance NGF secretion and remyelination of Schwann cells.^[^ ^33^, ^34^ ^]^ Bioceramics are biodegradable materials capable of simultaneously and slowly releasing various bioactive ions (e.g., Ca, Mg, Si, or Li) over an extended period. Certain types of bioceramics, such as hydroxyapatite, calcium silicate (CS), and akermanite (AT), have been utilized in bone regeneration, wound healing, and angiogenesis.^[^ ^35^, ^36^, ^37^, ^38^ ^]^ Therefore, the accumulated evidence on bioceramics regulating cell behavior suggests their potential to promote nerve regeneration in long‐gap PNI, although this possibility is yet to be explored.

**Scheme 1:**
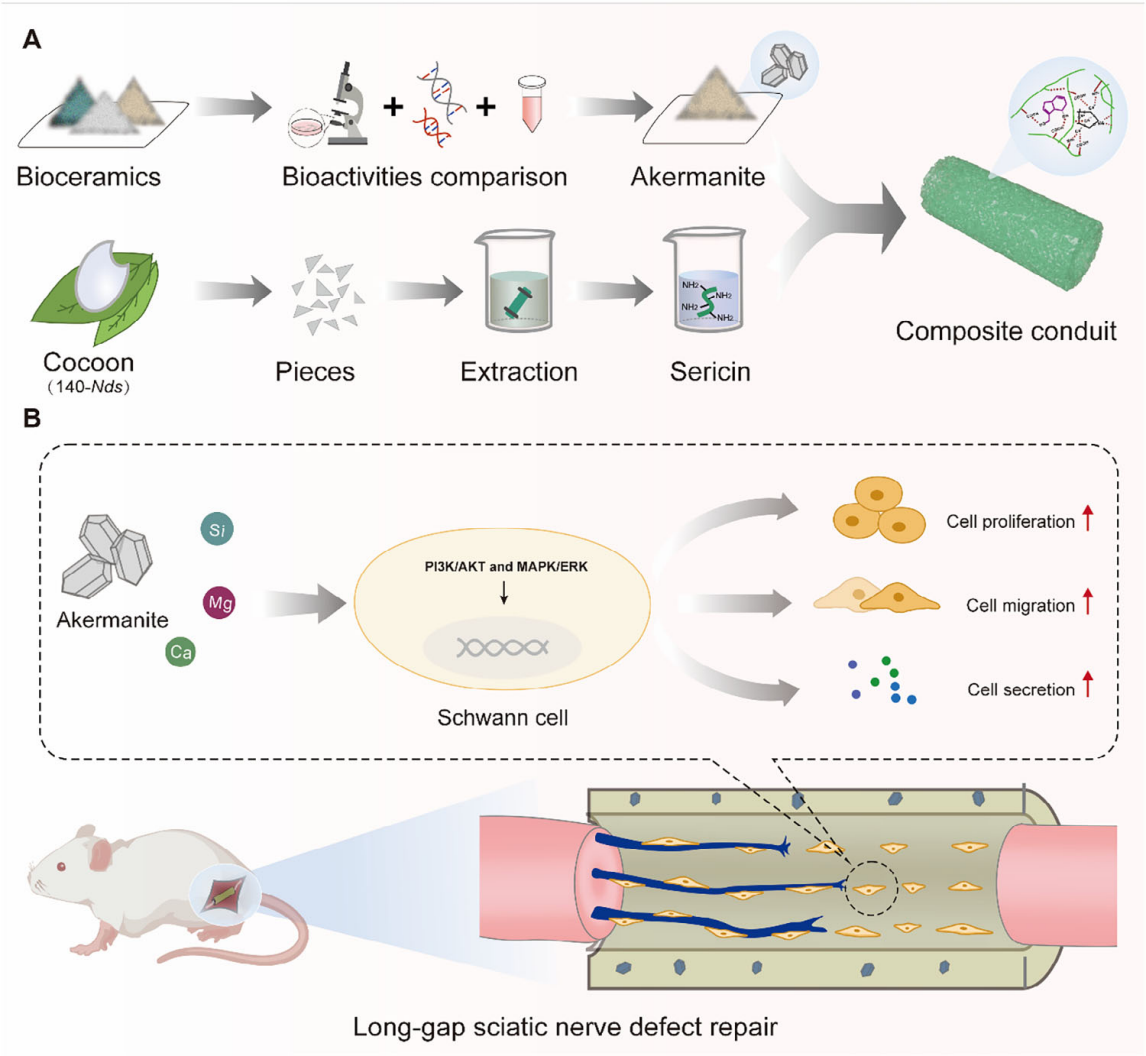
A) Schematic diagram illustrating the fabrication of bioceramic/SS nerve conduits. B) Schematic diagram illustrating that AT can enhance Schwann cell proliferation, migration, and secretion by activating PI3K/AKT and MAPK/ERK signaling, and AT‐SS composite conduit successfully repairs long‐gap sciatic nerve defects.

Silk sericin (SS), a natural biomaterial derived from silkworm cocoons, exhibits excellent cytocompatibility, biodegradability, and low immunogenicity, has been widely applied in skeletal muscle regeneration,^[^ ^39^ ^]^ wound healing,^[^ ^40^ ^]^ and myocardial repair.^[^ ^41^ ^]^ Recent studies have confirmed that SS possesses neurotrophic and neuroprotective bioactivities, promotes axonal extension, reduces neuronal apoptosis, and comprises functional groups for modification,^[^ ^42^, ^43^, ^44^, ^45^ ^]^ making it an ideal matrix for fabricating nerve conduits for PNI repair. In the current study (**Scheme** **1**), six bioceramics were compared, and AT was identified for its superior cytocompatibility, anti‐inflammatory properties, and neurotrophic factors regulation. The ability of AT to enhance the pro‐regenerative function of Schwann cells was further revealed. Moreover, AT and SS exerted synergistic effects on the pro‐regenerative behaviors of Schwann cells, given that SS promoted the release of Ca and Mg from AT. Regarding the repair of a 13‐mm limiting size PNI, the AT‐SS conduit effectively improved nerve structural and functional recovery compared with the commercial eton conduit, with behavioral restoration comparable with the “gold standard” autograft. This study addresses the limited biological activity of traditional conduits and furnishes a novel therapeutic strategy for long‐gap PNI with strong clinical translation potential.

## 2 Results and Discussion

### 2.1 Comparison of the Bioactivities of Various Bioceramics

**Figure 1:**
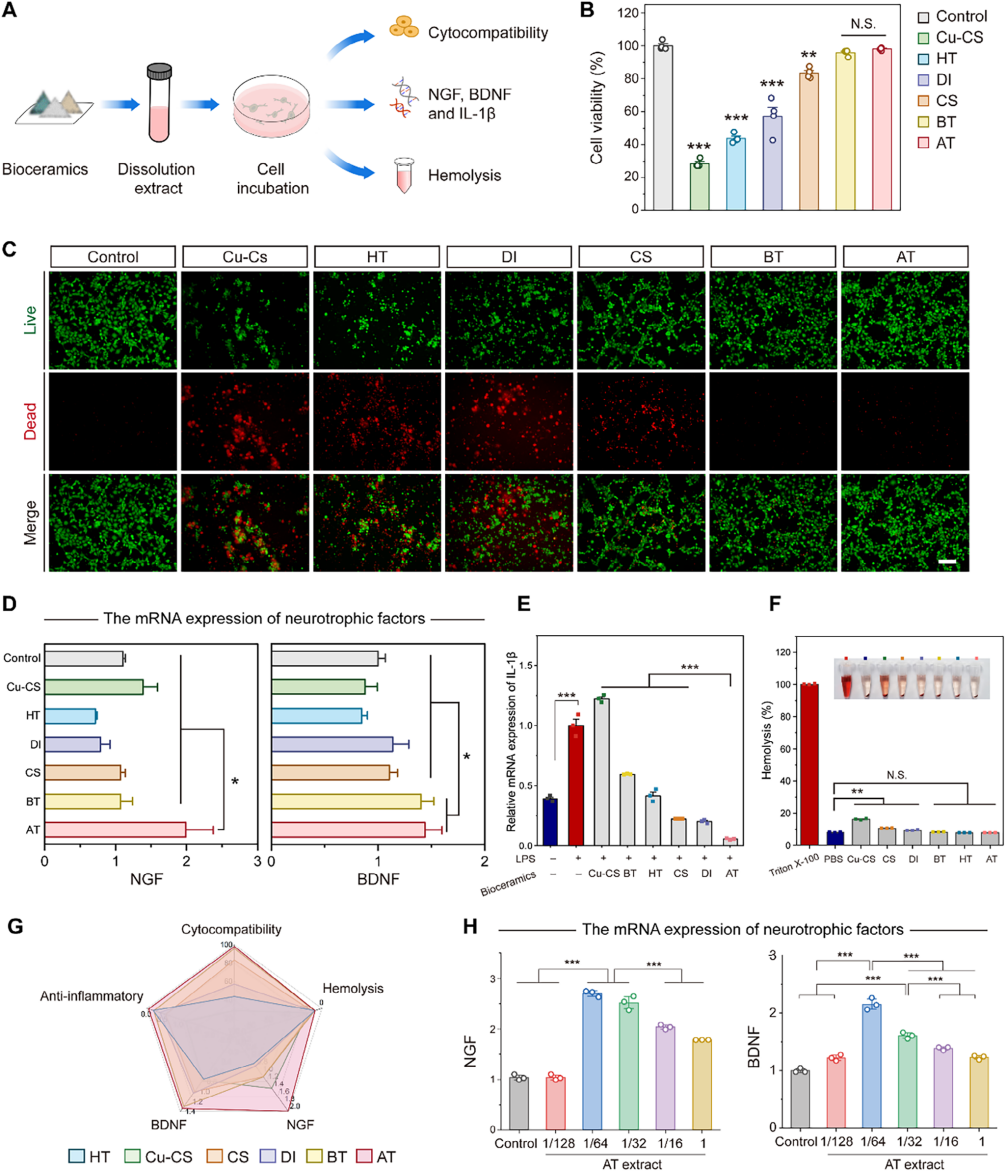
Bioactivity evaluation of the six bioceramics. A) Schematic diagram of testing the bioceramics’ bioactivities. B) The viability of RSC 96 cells cultured with original dissolution extracts of various bioceramics for 24 h (*n* = 4). C) Live and Dead staining images of RSC 96 cells treated with original dissolution extracts of various bioceramics for 24 h. Scale bar, 100 µm. D) Relative mRNA expression of NGF and BDNF in RSC 96 cells treated with original dissolution extracts of various bioceramics for 24 h (*n* = 3). E) Relative mRNA expression of IL‐1β in LPS‐stimulated RAW 264.7 cells treated with original dissolution extracts of different bioceramics (*n* = 3). F) The hemolysis assay of different bioceramic powders (*n* = 3). G) Comparison of the bioactivities of different bioceramics. H) Relative mRNA expression of NGF and BDNF in RSC 96 cells incubated with different dilutions of AT extracts for 24 h (*n* = 3). Data were presented as mean ± SD; *, *P* < 0.05; **, *P* < 0.01; ***, *P* < 0.001; N.S., not significant; ANOVA.

Based on their elemental compositions and structural features, several types of bioceramics are available, with some applied to repair diverse tissue injuries owing to their ability to regulate cell behaviors and “repair” cell function. For example, diopside (DI, MgCaSi₂O₆) could promote the proliferation and differentiation of osteoblastic‐like cells, enhancing bone repair.^[^ ^46^ ^]^ Recently, bioceramics including CS (CaSiO_3_), copper‐incorporated CS (Cu‐CS, Cu‐CaSiO_3_), Hardystonite (HT, Ca_2_ZnSi_2_O_7_), Bredigite (BT, Ca_7_MgSi_4_O_16_), and Akermanite (AT, Ca_2_MgSi_2_O_7_), have demonstrated efficacy in bone tissue repair, wound healing, and orbital reconstruction,^[^ ^47^, ^48^, ^49^, ^50^, ^51^ ^]^ while exhibiting the potential to repair nerve tissue.^[^ ^52^, ^53^, ^54^, ^55^, ^56^ ^]^ Accordingly, these six bioceramics were subjected to a series of experiments to assess their prospective application for peripheral nerve regeneration (**Figure** **1**A). To evaluate the cytocompatibility of these bioceramics, rat RSC 96 Schwann cells, a critical cell type that supports peripheral nerve regeneration, were incubated with the original dissolution extracts and subjected to CCK‐8 assay. Although the cell viability of AT and BT groups remained unchanged after a 24‐h incubation, it was significantly reduced in other bioceramic groups (Figure 1B), indicating the favorable cytocompatibility of AT and BT. Live and Dead cell staining revealed that the examined groups showed similar cytocompatibility (Figure 1C). The reduced cytocompatibility of Cu‐Cs and HT could be attributed to the excessive release of Cu and Zn ions during extraction, leading to cell death.^[^ ^57^, ^58^, ^59^, ^60^ ^]^ The decreased cytocompatibility of DI and CS may be due to the substantial release of Ca ions, resulting in the formation of Ca(OH)₂ and an increase in the pH, ultimately inducing cell death.^[^ ^61^, ^62^ ^]^ Given the critical roles of NGF and BDNF in promoting axon survival and extension, as well as remyelination after PNI,^[^ ^63^, ^64^, ^65^ ^]^ we investigated whether these bioceramics can impact the Schwann cell secretion of these two neurotrophic factors. Quantitative real‐time PCR analysis revealed that AT simultaneously enhanced the mRNA expression of NGF and BDNF, whereas BT only upregulated the mRNA expression of BDNF (Figure 1D). Treatment with the other four bioceramics did not notably upregulate the expression of BDNF or NGF. These results indicate that AT and BT enhance the secretion of neurotrophic factors. Furthermore, AT extracts significantly enhanced NGF expression in RSC 96 cells compared with other bioceramics (Figure S1, Supporting Information). As excessive inflammatory responses could inhibit nerve regeneration,^[^ ^66^, ^67^ ^]^ the expression of IL‐1β mRNA, a crucial pro‐inflammatory cytokine,^[^ ^68^ ^]^ was evaluated in the lipopolysaccharide (LPS)‐stimulated RAW 264.7 cells treated with various bioceramic extracts. Five of these bioceramics could significantly inhibit mRNA expression of IL‐1β, with AT demonstrating the most pronounced inhibitory effect (Figure 1E). Furthermore, AT could significantly suppress the mRNA expression of IL‐6 and TNF‐α in LPS‐stimulated RAW 264.7 cells (Figure S2, Supporting Information). These results indicate that AT exerts the strongest anti‐inflammatory activity among the six bioceramics. Additionally, BT, HT, and AT showed good hemocompatibility as evidenced by the results of the hemolysis assay (Figure 1F). Based on the cytocompatibility, neurotrophic factors regulation, anti‐inflammatory ability, and hemocompatibility (Figure 1G), AT was identified as the most appropriate bioceramic for peripheral nerve regeneration. Because bioactive ions released from bioceramics in vivo undergo gradual dilution through tissue fluid diffusion, AT extracts were prepared at different dilutions (1, 1/16, 1/32, 1/64, and 1/128) for RSC 96 cell culture to better simulate the physiological scenario. Notably, the 1/64 and 1/32 dilutions significantly enhanced the expression of NGF and BDNF (Figure 1H). Accordingly, these two dilution ratios were selected for subsequent experiments.

### 2.2 AT Simultaneously Enhances RSC 96 Schwann Cell Proliferation, Migration, and Secretion Behaviors In Vitro

**Figure 2:**
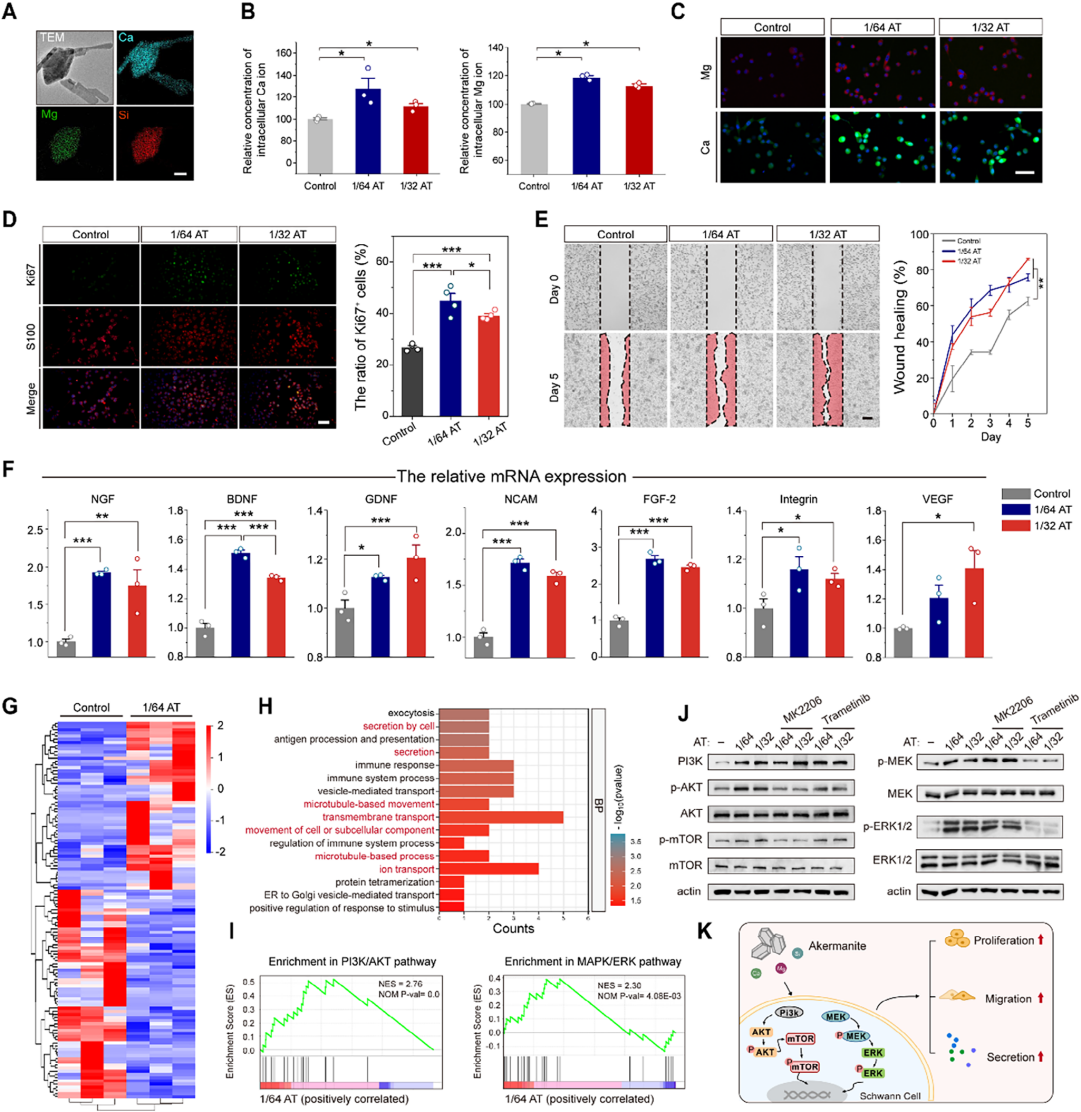
AT enhances the proliferation, migration, and secretion of RSC 96 Schwann cells. A) TEM and EDS analyses of AT. Scale bar, 100 nm. B) Relative concentration of intracellular Ca and Mg ions in RSC 96 cells incubated with 1/64 and 1/32 diluted AT extracts for 24 h (*n* = 3). C) Results of intracellular Mg and Ca ions detection using ethyl benzo[6,7]‐4‐*oxo*‐4H‐quinolizine‐3‐carboxlate and Fura‐2 AM. Mg is indicated in red (fake color), and Ca is indicated in green. Scale bar, 20 µm. D) Anti‐Ki67 and anti‐S100 staining images of RSC 96 cells incubated with diluted AT dissolution extracts for 48 h. Scale bar, 200 µm. The ratio of Ki67‐positive cells in the indicated groups was quantified and shown on the right (*n* = 4). E) Wound healing images of RSC 96 cells treated with diluted AT dissolution extracts in Scratch tests. The boundaries of scratch wounds are indicated by black dashed lines, and the cell‐migrating regions are marked in red. Scale bar, 200 µm. The ratios of wound healing in the indicated groups were calculated and placed on the right (*n* = 3). F) Relative mRNA levels of NGF, BDNF, GDNF, NCAM, FGF‐2, integrin, and VEGF in RSC 96 cells treated with diluted AT dissolution extracts for 24 h (*n* = 3). G) Heatmap of differentially expressed genes between the control and 1/64 AT groups. H) Bar graphs of GO enriched in the biological process (BP). I) GSEA plots of gene sets associated with PI3K/AKT and MAPK/ERK signaling pathways. Each graph shows the enrichment plots of gene expression in the 1/64 AT group compared with that in the control group. NES, normalized enrichment score; NOM P‐val, nominal *P*‐value. J) Expression of key protein kinases involved in the PI3K/AKT pathway (left) and the MAPK/ERK pathway (right) was tested in RSC 96 cells treated with diluted AT extracts in the presence or absence of AKT inhibitor (MK2206) and the MEK inhibitor (Trametinib). K) Schematic illustration of AT enhances the proliferation, migration, and secretion of Schwann cells. Data were presented as mean ± SD; *, *P* < 0.05; **, *P* < 0.01; ***, *P* < 0.001; ANOVA.

As a Ca‐, Mg‐, and Si‐enriched bioceramic material, AT could release bioactive ions during extraction (**Figures** **2**A and S3, Supporting Information). Treatment with diluted AT extracts significantly enhanced intracellular Ca and Mg ion levels in RSC 96 cells (Figure 2B,C). However, the absence of substantial intracellular Si ion accumulation suggests either a lack of specific Si ion channels or limited Si transport mechanisms in Schwann cells. To determine whether AT influences the pro‐regenerative behaviors of Schwann cells,^[^ ^69^, ^70^ ^]^ the following tests were performed to examine the cell proliferation, migration, and secretion of RSC 96 cells treated with 1/64 and 1/32 diluted AT extracts. To evaluate the proliferation of Schwann cells, anti‐Ki67 (a marker of cellular proliferation^[^ ^71^ ^]^) immunofluorescence staining and CCK‐8 assays were conducted after a 48‐h incubation. The Ki67‐positive cell ratio and cell viability of the 1/64 and 1/32 AT groups were significantly higher than those in the control groups (Figures 2D and S4, Supporting Information), indicating that AT promotes the proliferation of RSC 96 Schwann cells. The migration behavior of Schwann cells was evaluated using both Scratch and Transwell assays. The results of the Scratch assay revealed that the 1/64 and 1/32 AT groups had faster wound healing rates than the control group (Figures 2E and S5, Supporting Information). Moreover, improved cell migration was confirmed by Transwell assays (Figure S6, Supporting Information). To evaluate the expression of nerve regeneration‐associated factors, seven key genes, including NGF, BDNF, GDNF, NCAM, FGF‐2, integrin, and VEGF, were detected in AT‐treated RSC 96 cells. Both the 1/64 and 1/32 AT groups showed significantly upregulated mRNA levels of these genes (Figure 2F), suggesting that AT enhances the secretion function of RSC 96 Schwann cells. Together, these results indicate that AT can simultaneously enhance pro‐regenerative functions of Schwann cells, including cell proliferation, migration, and secretion.

RNA sequencing was performed to determine the underlying mechanism through which AT regulates the pro‐regenerative behaviors of Schwann cells. AT significantly altered the molecular phenotypes of RSC 96 Schwann cells (Figure 2G), and gene ontology (GO) analysis identified differentially expressed genes associated with cellular secretion, microtubule motor activity, and ion transport (Figures 2H and S7, Supporting Information). Gene set enrichment analysis (GSEA) further revealed significant enrichment in PI3K/AKT and MAPK/ERK signaling pathways (Figure 2I), which play pivotal roles in regulating Schwann cell proliferation, migration, and secretion.^[^ ^72^, ^73^ ^]^ The above analysis suggests that AT bioceramic regulates the pro‐regenerative behaviors of RSC 96 Schwann cells by activating these two signaling pathways. In support of this, the levels of key protein kinases involved in the PI3K/AKT signaling pathway (PI3K, p‐AKT, and p‐mTOR) and the MAPK/ERK signaling pathway (p‐MEK and p‐ERK) were significantly elevated in the 1/64 and 1/32 AT groups compared with those in the control group. Notably, these elevated levels were markedly suppressed by the AKT inhibitor MK2206 and the MEK inhibitor trametinib (Figures 2J and S8, Supporting Information). Inhibition of the PI3K/AKT and MAPK/ERK signaling pathways blocked the AT‐mediated enhancement of RSC 96 cells proliferation, migration, and nerve regeneration‐associated factors expression (Figure S9, Supporting Information). Collectively, these results suggest that AT bioceramic can improve the pro‐regenerative behaviors of RSC 96 Schwann cells by activating the PI3K/AKT and MAPK/ERK signaling pathways (Figure 2K).

### 2.3 Characterization of AT and AT‐SS Composite Conduits

**Figure 3:**
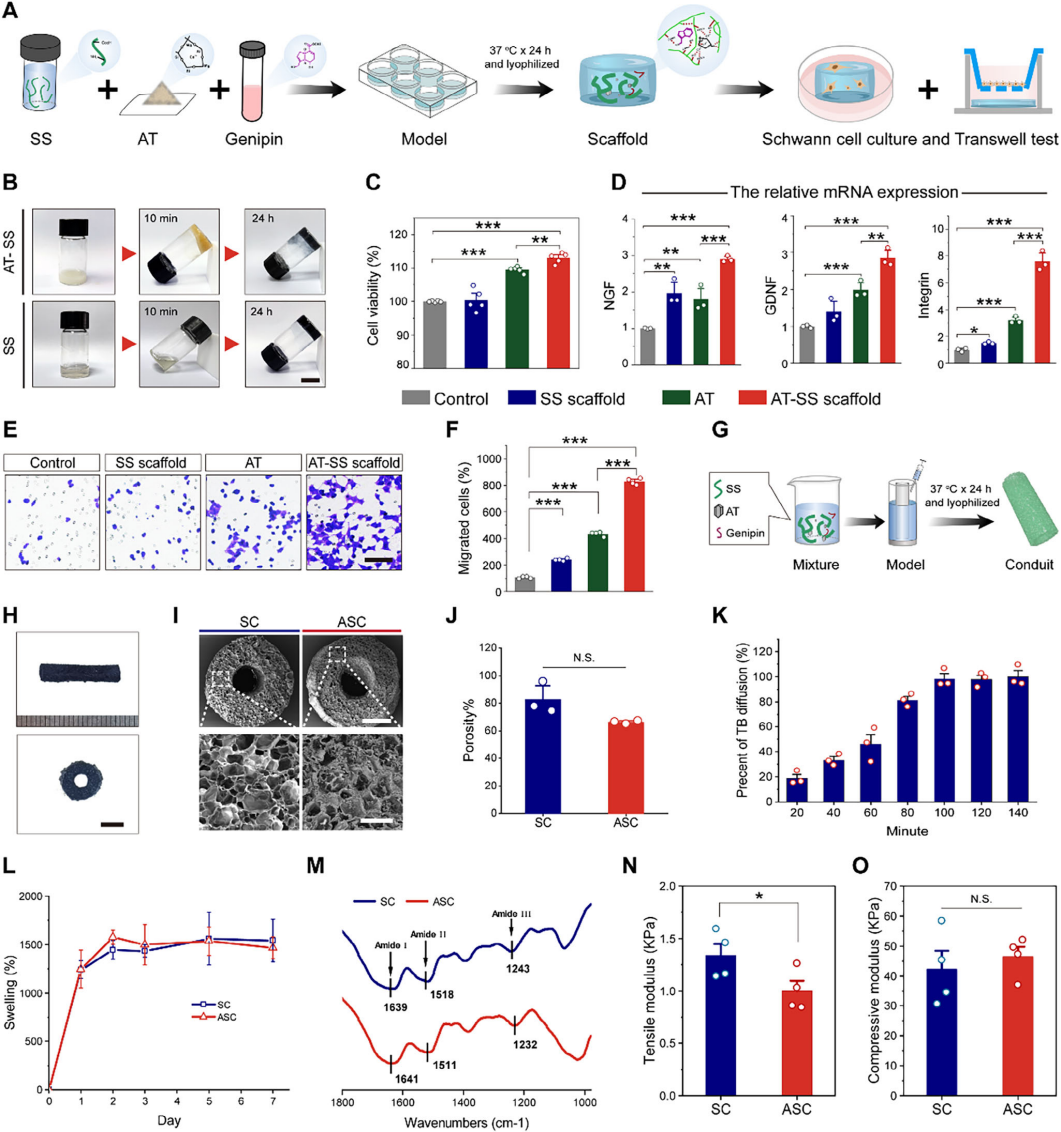
Characterization of the AT‐SS scaffold and composite conduit. A) Schematic diagram illustrating the fabrication of the AT‐SS scaffold and bioactivity examination process. B) Photographs showing the gelation process of AT‐SS and SS scaffolds. Scale bar, 1 cm. C) Cell viability of RSC 96 cells cultured with AT‐SS scaffold for 48 h (*n* = 5). D) The relative expression of NGF, GDNF, and integrin mRNA in RSC 96 cells cultured with AT‐SS scaffold for 24 h (*n* = 3). E,F) Images of migrated RSC 96 cells incubated with AT‐SS scaffold (E) in Transwell tests and the ratio of migrated cells (F) (*n* = 4). Scale bar, 200 µm. G) Schematic diagram illustrating the ASC fabrication process. H) Photographs of ASC from vertical and horizontal views. Scale bar, 2 mm. I) SEM images of the cross‐section of SC and ASC. The enlarged regions are boxed with white dashed lines. Scale bars, 1 mm for upper panels, 200 µm for lower panels. J) Porosity results of SC and ASC (*n* = 3). K) The TB diffusion percent of ASC at different time points (*n* = 3). L) Swelling dynamic behaviors of SC and ASC (*n* = 3). M) FTIR spectra of SC and ASC. N) The tensile modulus of ASC and SC (*n* = 4). O) The compressive modulus of ASC and SC (*n* = 4). Data were presented as mean ± SD; *, *P* < 0.05; **, *P* < 0.01; ***, *P* < 0.001; N.S., not significant; ANOVA or Student's *t*‐tests.

Owing to its excessive mechanical strength and fragility, AT powder must be composited with other materials to fabricate nerve conduits. Given the excellent bioactivity of SS and its abundant functional groups for modification,^[^ ^42^, ^43^, ^44^, ^45^ ^]^ SS is an ideal matrix material for compositing with AT to construct nerve conduits. To test this hypothesis, an AT‐SS composite scaffold was fabricated by incorporating AT into genipin‐crosslinked SS and used to culture RSC 96 cells to evaluate their proliferation, secretion, and migration behaviors (**Figure** **3**A). The addition of AT to the SS and genipin solutions significantly accelerated the gelation process (Figure 3B), likely because of the chelation of Ca and Mg ions released from AT with SS.^[^ ^74^, ^75^, ^76^ ^]^ Unlike the Li─Mg─Si bioceramic nerve conduit, which reduces Schwann cells viability,^[^ ^77^ ^]^ both SS and AT‐SS scaffolds demonstrated excellent cytocompatibility, as evidenced by the GFP‐RSC 96 cells maintaining high viability and proliferation activity in a 48‐h 3D culture experiment (Figure S10, Supporting Information). Additionally, long‐term culture did not induce significant death in PC 12 cells (Figure S11, Supporting Information), further supporting this notion. Notably, the AT‐SS scaffold group exhibited significantly higher cell viability than the AT group, whereas the SS scaffold group showed no notable change (Figure 3C). The AT‐SS scaffold distinctly enhanced nerve regeneration‐associated factor expression, with upregulated expression of NGF, GDNF, and integrin mRNA, compared with the AT and SS scaffold groups (Figure 3D). Expression levels of genes such as BDNF, FGF‐2, NCAM, and VEGF were upregulated in the AT‐SS group compared with those in the control group (Figure S12, Supporting Information). The AT‐SS scaffold significantly enhanced cell migration compared with the AT and SS scaffold groups, with cell migration increasing by 140.5%, 335.3%, and 729.2% in the SS, AT, and AT‐SS scaffold groups, respectively (Figure 3E,F). Together, these results indicate that AT and SS could synergistically enhance the pro‐regenerative functions of Schwann cells. To explore the underlying mechanisms, we analyzed the ion release profiles of the AT‐SS scaffolds, revealing that the release of Ca and Mg ions was significantly accelerated, whereas that of Si ions remained unchanged (Figure S13, Supporting Information). This finding could be attributed to the acidic microenvironment formed around AT by abundant acidic amino acids in SS (e.g., Asp and Glu), which accelerates the release of cations.^[^ ^78^, ^79^, ^80^ ^]^ This accelerated ion release could rapidly initiate the AT‐mediated enhancement of Schwann cell pro‐regenerative behavior. Chelated Ca and Mg ions reportedly exhibit enhanced biological activity.^[^ ^81^, ^82^, ^83^ ^]^ Collectively, this accelerated ion release and ion chelation may jointly contribute to the synergistic effects of AT and SS on pro‐regenerative behaviors of RSC 96 Schwann cells.

After injecting a mixture of SS, AT, and genipin into a mold and incubating at 37 °C for 24 h, AT‐SS composite conduits were successfully formulated via lyophilization and termed ASC (Figure 3G). ASC was dark blue with hollow tube structures, 15 mm in length, 0.75 mm in wall thickness, 1.5 mm in inner diameter, and with a wall thickness to inner diameter ratio of 1:2 (Figure 3H). SEM images revealed that this composite conduit possessed highly porous microstructures (Figure 3I), with a porosity of 66.3%, which was comparable to that of the sericin conduit (SC) (Figure 3J). Using toluidine blue (TB; 373.9 Da) as a representative low‐molecular‐weight indicator,^[^ ^84^ ^]^ the ASC demonstrated efficient permeability, achieving 50% TB diffusion within 60 min (Figure 3K). This highly porous microstructure indicates that ASC could facilitate the exchange of metabolites and nutrients during nerve regeneration. Following immersion in PBS for 24 h, the conduits demonstrated a rapid 15‐fold increase in volume, ultimately reaching a state of equilibrium (Figure 3L), which prevented the conduits from compressing the regenerated nerve tissues. This composite conduit had three characteristic Fourier transform infrared (FTIR) absorption bands, amide I (1641 cm^−1^), amide II (1511 cm^−1^), and amide III (1232 cm^−1^) (Figure 3M), which were the special absorption peaks of SS.^[^ ^43^ ^]^ Accordingly, incorporating AT does not substantially transform the secondary structure of SS. Furthermore, conduits must possess appropriate mechanical properties to ensure their neurorepair performance.^[^ ^85^ ^]^ While polymer materials, such as PLLA conduits with a tensile strength of 64.3–69.8 MPa,^[^ ^86^ ^]^ exhibited excessive mechanical strength that may induce nerve tissue damage, this SS‐based conduit displayed a markedly lower tensile modulus. The tensile modulus of ASC was 1.0 kPa, lower than that of SC, making it more compatible with nerve tissues (Figures 3N and S14, Supporting Information). The compressive modulus (Figures 3O and S15, Supporting Information) suggests that this conduit could tolerate compression from surrounding tissues. The excellent flexibility of this hybrid conduit was further confirmed by the slight decrease in diameter after 1000‐cycle compression (Figure S16, Supporting Information). Collectively, the mechanical properties of ASC render it suitable for nerve repair.

### 2.4 ASC Promotes the Proliferation, Migration, and Secretion of Schwann Cells In Vivo

**Figure 4:**
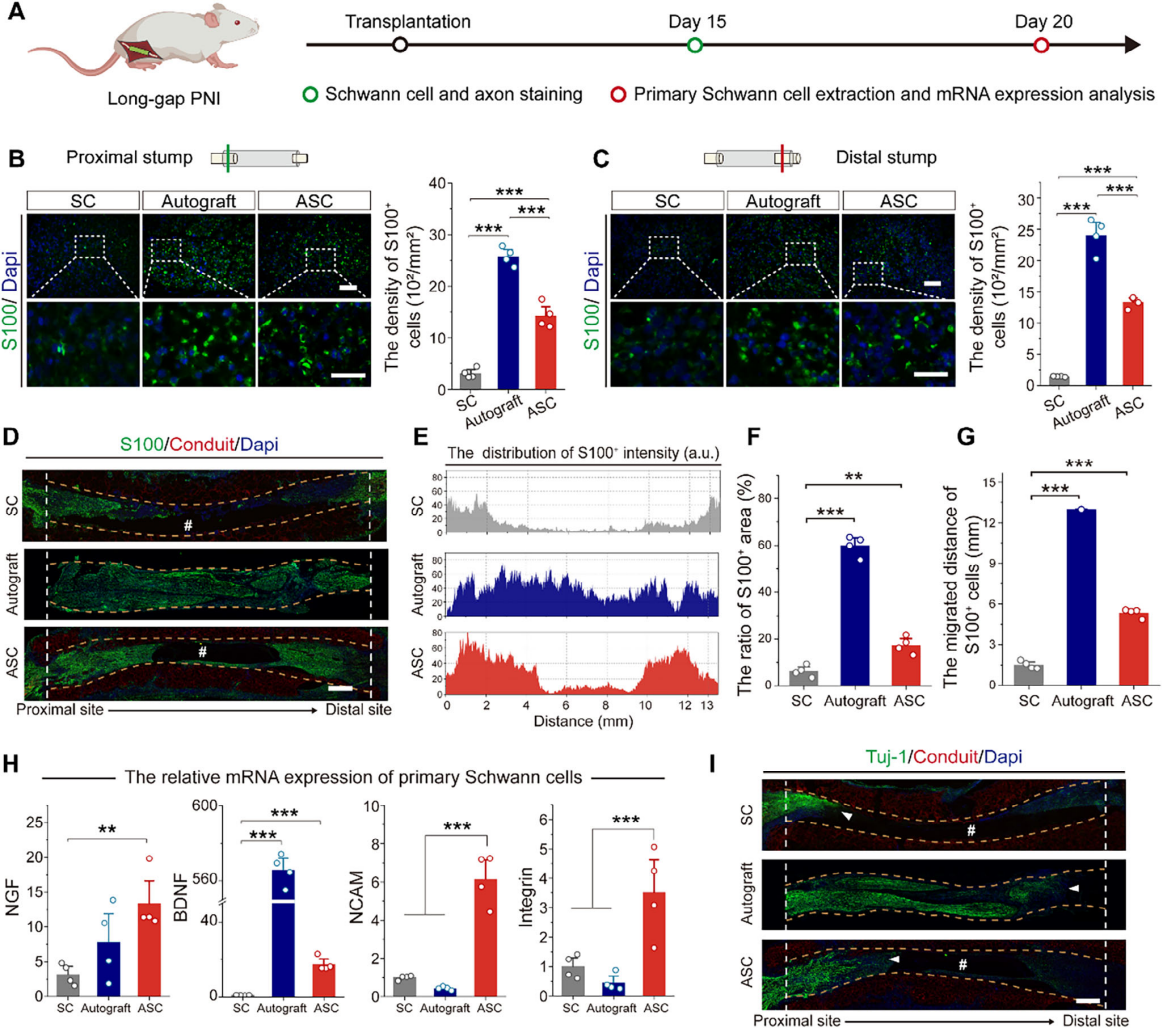
In vivo evaluation of ASC‐mediated regulation of the pro‐regenerative behaviors of Schwann cells. A) Experimental flowchart for in vivo examination of the regulating ability of ASC. B,C) Fluorescence images of the Schwann cells (S100, green) on the transverse sections of proximal (B) and distal stumps (C) of damaged nerves 15 days post‐transplantation. Conduits show red self‐fluorescence. Enlarged regions are boxed with white dashed lines, and the density of S100‐positive cells is calculated and placed on the right. Four rats in each group. Scale bars, 50 µm for upper panels, 25 µm for lower panels. D) Fluorescence images of Schwann cells (S100, green) on the longitudinal sections of conduits 15 days post‐transplantation. The pound sign indicates the conduit lumen. Scale bar, 1 mm. E) The distribution of S100‐positive intensity correlating with length variations in (D). F) The ratio of S100‐positive area within the lumen of conduits and regenerated nerve. Four rats in each group. G) The migratory distance of S100‐positive cells from proximal sites. Four rats in each group. H) The relative mRNA expression of NGF, BDNF, NCAM, and integrin of primary Schwann cells after 20‐day transplantation. Four rats in each group. I) Fluorescence images of axons (Tuj‐1, green) in longitudinal sections of conduits 15 days post‐implantation. The pound sign indicates the conduit lumen, and the white arrowheads indicate the axonal extension front. Scale bar, 1 mm. Data were presented as mean ± SD; **, *P* < 0.01; ***, *P* < 0.001; ANOVA.

Given the differences in proteomic and functional characteristics, the RSC 96 cell line fails to fully replicate the behaviors of primary Schwann cells.^[^ ^87^ ^]^ To bridge this gap and extend our findings from the RSC 96 cell line to primary Schwann cells, we comprehensively evaluated the proliferation, migration, and secretion behaviors of Schwann cells in rat PNI models following ASC implantation (**Figures** **4**A and S17, Supporting Information). Fifteen days post‐transplantation, anti‐S100 immunofluorescence staining was performed on the transverse sections at the proximal and distal stumps of damaged nerves to visualize the Schwann cells (Figure 4B,C). The S100‐positive cell densities of ASC groups at the proximal and distal sites were significantly higher than those of the SC group, indicating that ASC could promote Schwann cell proliferation. In addition, the ASC group demonstrated significant Schwann cell migration from the nerve stumps to the lumen of conduits (Figure 4D,E), suggesting that ASC dramatically promotes the Schwann cell migration for long‐gap PNI repair. This was further confirmed by the increased S100‐positive area and longer migration distance in the ASC group than in the SC group (Figure 4F,G). Primary Schwann cells were extracted to evaluate the mRNA expression of nerve regeneration‐associated factors 20 days post‐transplantation (Figure S18, Supporting Information). Compared with SC group, the ASC group demonstrated significantly upregulated mRNA expression of NGF, BDNF, NCAM, and integrin (Figure 4H). In particular, the NCAM and integrin levels of ASC group were higher than those in the autograft group, probably because the expression of these two cytokines was more sensitive to the bioactive ions Ca and Mg.^[^ ^88^, ^89^, ^90^ ^]^ Collectively, these results suggest that ASC could substantially promote the expression of regeneration‐associated factors in Schwann cells after long‐gap PNI repair. Notably, gene expression levels of FGF‐2 and VEGF showed no obvious change compared to the SC group (Figure S19, Supporting Information), probably because these genes typically demonstrate high expression within 2–3 days after damage, and rapidly return to normal levels.^[^ ^91^, ^92^, ^93^ ^]^ Moreover, the ASC group showed significantly longer axon extension than the SC group 15 days post‐implantation (Figure 4I), indicating that ASC markedly facilitates axonal sprouting. Taken together, ASC could enhance the proliferation, migration, and secretion of Schwann cells for long‐gap limiting size PNI repair, which further promotes axonal extension during regeneration.

### 2.5 ASC Enhances the Histological Reconstruction of Long‐Gap Limiting Size PNI

**Figure 5:**
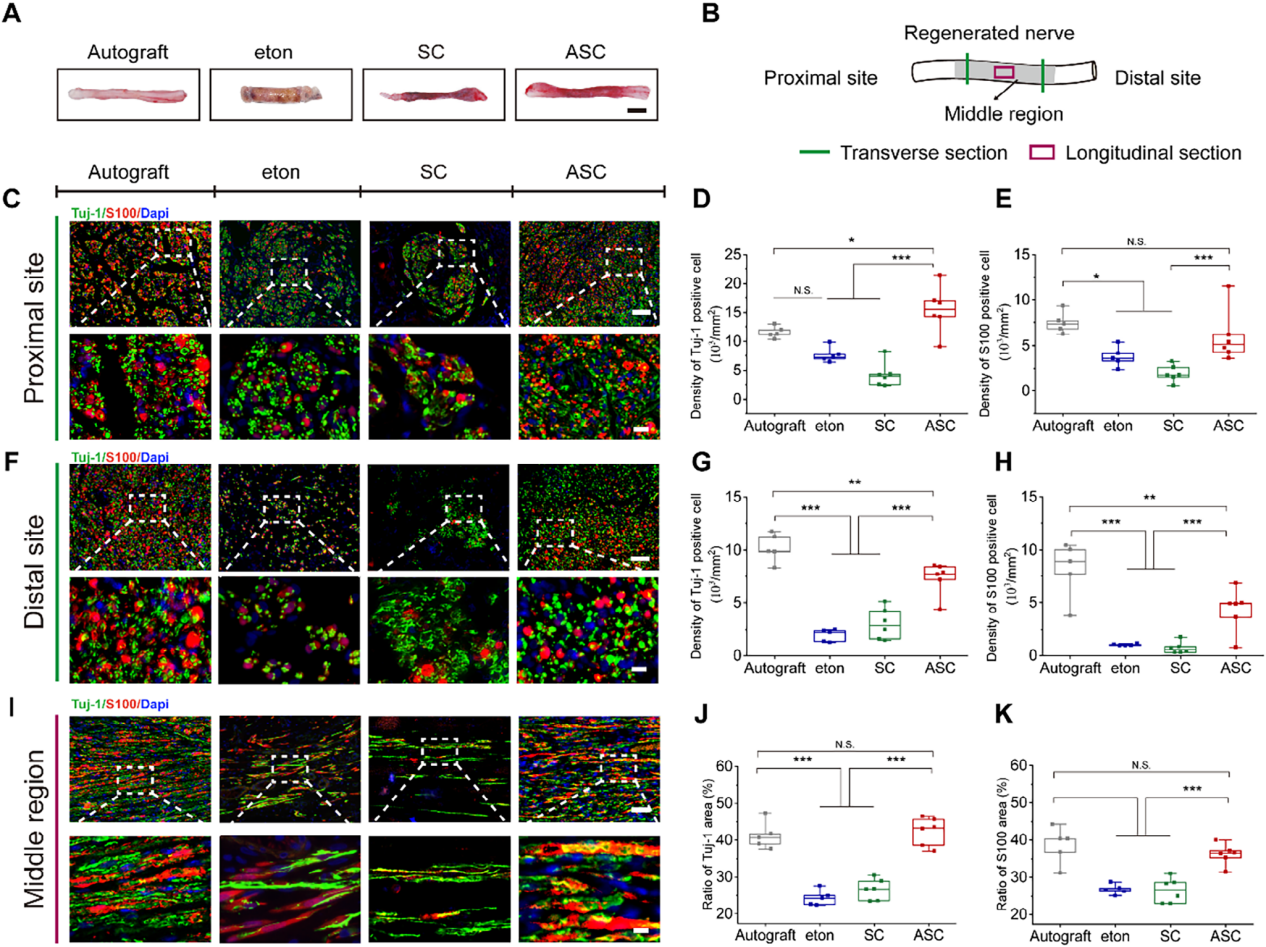
Histological evaluation of regenerated sciatic nerves following a 14‐week implantation. A) Photographs of regenerated sciatic nerves. Scale bar, 2 mm. B) Schematic illustrates the acquisition of nerve tissues in transverse and longitudinal sections. C) Fluorescence images of axons (Tuj‐1, green) and Schwann cells (S100, red) of transverse sections at the proximal site of regenerated nerves. The enlarged regions are boxed with white dotted lines. Scale bars, 50 µm for upper panels, 10 µm for lower panels. D,E) The densities of Tuj‐1‐positive cells D) and S100‐positive cells E) in proximal sites. Six rats in the SC and ASC groups and five rats in the autograft and eton groups. F) Fluorescence images of axons (Tuj‐1, green) and Schwann cells (S100, red) of transverse sections at the distal site of regenerated nerves. The enlarged regions are boxed with white dotted lines. Scale bars, 50 µm for upper panels, 10 µm for lower panels. G,H) The densities of Tuj‐1‐positive cells (G) and S100‐positive cells (H) at distal sites. Six rats in the SC and ASC groups and five rats in the autograft and eton groups. I) Fluorescence images of axons (Tuj‐1, green) and Schwann cells (S100, red) of longitudinal sections in the middle section of regenerated nerves. The enlarged regions are boxed with white dotted lines. Scale bars, 50 µm for upper panels, 10 µm for lower panels. J,K) The ratios of Tuj‐1‐positive areas (J) and S100‐positive areas (K) in the middle regions. Six rats in the SC and ASC groups and five rats in the autograft and eton groups. Data were presented as mean ± SD; *, *P* < 0.05; **, *P* < 0.01; ***, *P* < 0.001; N.S., not significant; ANOVA.

To evaluate the therapeutic outcomes of ASC, histological analysis of the regenerated nerve tissues was performed at 14 weeks post‐transplantation. A recently approved commercial nerve conduit eton (Medical Device Registration Certificate No. 20 203 130 898; NMPA, 2020) was used for comparison. The regenerated nerves in the ASC group were covered with capillaries and showed no obvious scar tissue formation (**Figure** **5**A), indicating the favorable compatibility of ASC. Furthermore, anti‐Tuj‐1 and anti‐S100 staining were performed at various sites to assess regenerated axons and Schwann cells (Figure 5B). At the proximal sites, the ASC group showed a higher axon proportion and similar Schwann cell density compared with the autograft group, while both parameters were significantly higher than those in the SC groups (Figure 5C–E). The increased axon proportion may result from the combined effects of activated Schwann cells and the Mg ions released from AT.^[^ ^33^ ^]^ At the distal sites, the ASC group exhibited significantly higher axons and Schwann cells densities than the SC group, and 3.8‐fold and 4.3‐fold higher densities than the eton group, respectively (Figure 5F–H). Although the ratio of regenerated axons in the ASC group exceeded that of both the eton and SC groups, it was lower than that in the autograft group. Notably, none of these treatments achieved the levels observed in normal intact nerves (Figure S20, Supporting Information). In the middle region, the ratios of oriented axons and Schwann cells in the ASC group were comparable to those in the autograft group, and obviously higher than those in the eton and SC groups (Figure 5I–K). These results indicate that ASC could substantially restore axons and Schwann cells to repair long‐gap limiting size PNI. However, the axon regeneration in the ASC group at the distal site was lower than that in the autograft group, likely resulting from insufficient microstructural support for axon growth. The reduced Schwann cell density in the ASC group may be due to Schwann cell atrophy caused by delayed axonal contact.^[^ ^94^, ^95^, ^96^ ^]^

### 2.6 ASC Enhances Remyelination of Regenerated Sciatic Nerves

**Figure 6:**
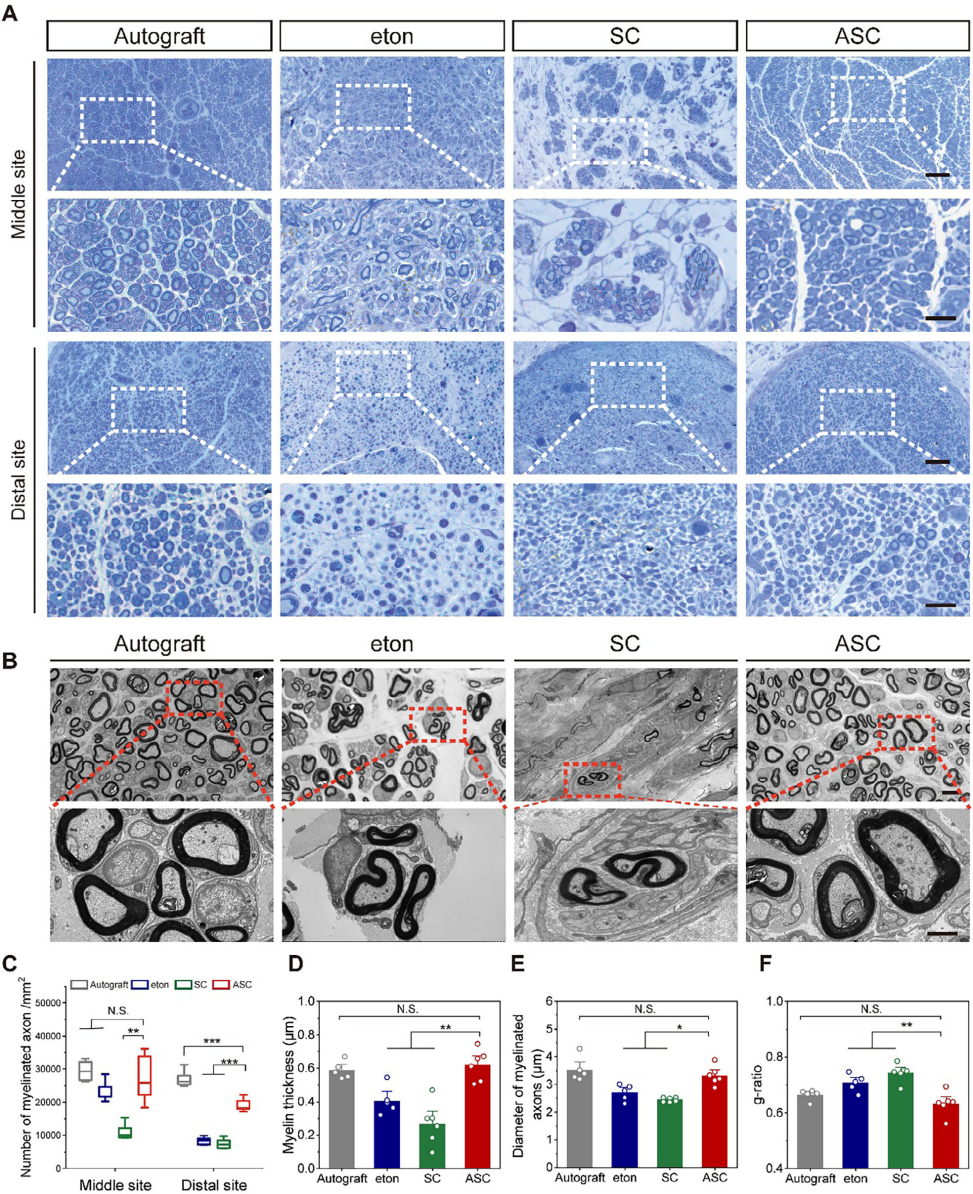
Histological assessment of regenerated myelin sheaths. A) TB staining of regenerated nerve sections at the middle and distal sites. The enlarged regions are boxed with white dotted lines. Scale bars, 100 µm for upper panels, 20 µm for lower panels. B) TEM images of myelin sheath at the distal site of regenerated nerve. Enlarged regions are boxed with red dashed lines. Scale bars, 5 µm for upper panels, 2 µm for lower panels. C) Percentage of myelinated axons in indicated groups. Six rats in the SC and ASC groups and five rats in the autograft and eton groups. D–F) The myelin sheath thickness (D), diameter of myelinated axons (E), and g‐ratio (F) of regenerated nerves in the indicated groups. Six rats in the SC and ASC groups and five rats in the autograft and eton groups. Data were presented as mean ± SD; *, *P* < 0.05; **, *P* < 0.01; ***, *P* < 0.001; N.S., not significant; ANOVA.

Functional restoration of nerve regeneration relies on axonal remyelination.^[^ ^97^ ^]^ To evaluate myelin regeneration, TB staining was performed at the middle and distal sites of regenerated nerves (**Figure** **6**A). At the middle site, the myelinated axon density of the ASC group was comparable to that of the autograft group and significantly higher than that of the SC group. At the distal site, the myelinated axon density of the ASC group remained significantly higher than that of the SC group, and was 2.3‐fold higher than eton group (Figure 6C). Based on TEM analysis, the ASC and autograft groups exhibited myelinated nerve fiber clusters at the distal site of the regenerated nerve, which were sparse in the eton and SC groups (Figure 6B). The myelin thickness and myelinated axon diameter of the ASC group were comparable to the autograft group and significantly higher than those of eton and SC groups, respectively (Figure 6D,E). The ASC group had a g‐ratio, an index reflecting the function and structure of nerve myelination,^[^ ^98^ ^]^ of 0.63, which was significantly lower than the eton and SC groups and comparable to the autograft group (0.66), approaching the optimal theoretical value of 0.6^[^ ^99^ ^]^ (Figure 6F). These results suggest that ASC could enhance the remyelination of regenerated nerves in long‐gap limiting size PNI to a level comparable to that of autografts.

### 2.7 ASC Enhances the Functional Restoration of Long‐Gap Limiting Size PNI

**Figure 7:**
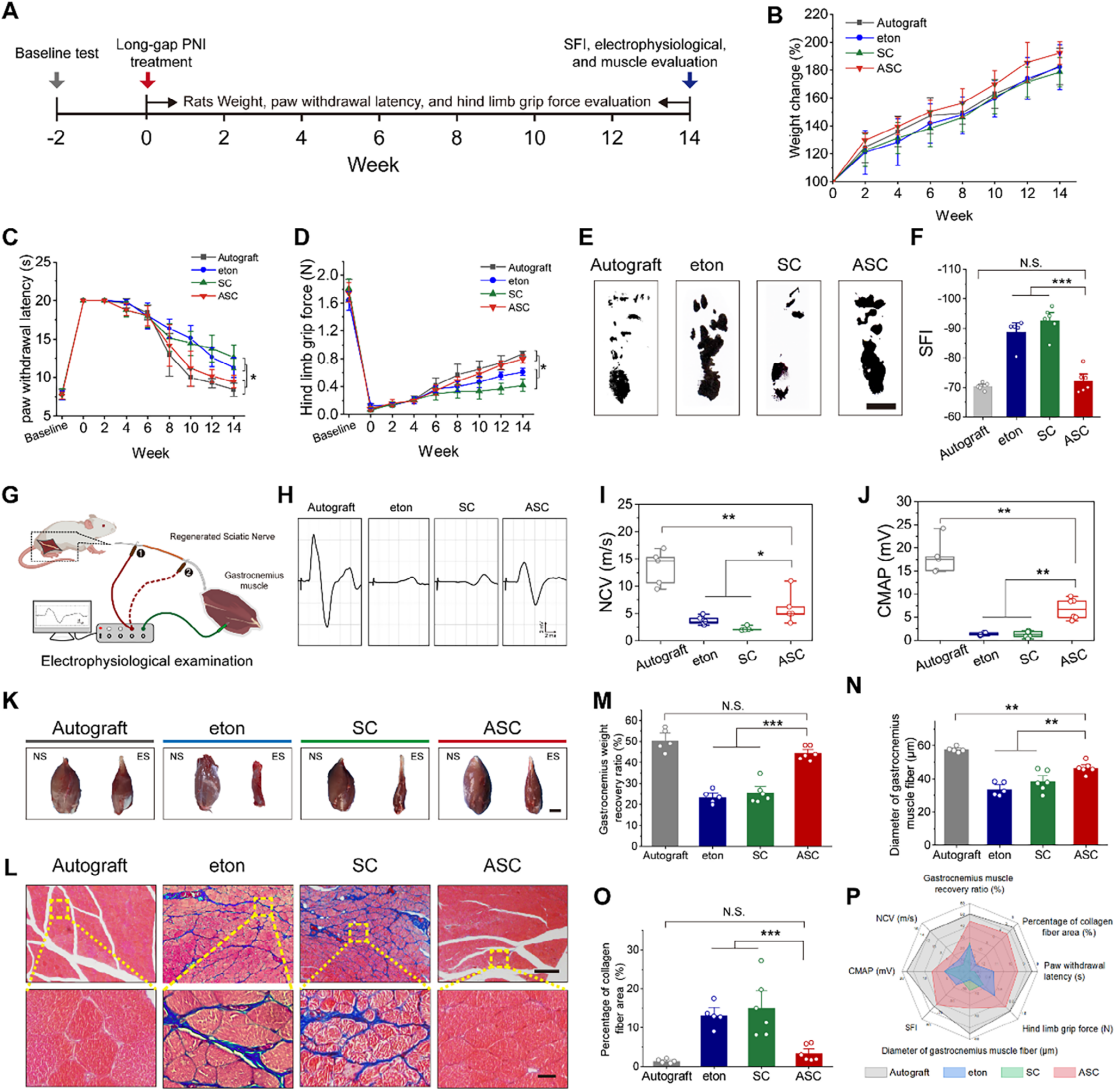
Functional examination and gastrocnemius muscle assessments after a 14‐week implantation. A) Experimental flowchart of neural function assessments at different time points post‐treatment. B–D) The weight change (B), paw withdrawal latency (C), and hind limb grip force examination (D) of rats at different time points post‐treatment. Six rats in the SC and ASC groups and five rats in the autograft and eton groups. E,F) Photographs of hind‐paw footprints (E) and SFI analysis (F) of rats 14 weeks after treatment. Scale bar, 1 cm. Six rats in the SC and ASC groups and five rats in the autograft and eton groups. G) Illustration of electrophysiological examination. H) Representative patterns of compound muscle action potential in the autograft, eton, SC, and ASC groups. I,J) The quantitative analysis of NCV (I) and CMAP (J) of rats 14 weeks after treatment. Six rats in the SC and ASC groups and five rats in the autograft and eton groups. K) Photographs of gastrocnemius muscles from the normal site (NS) and experimental site (ES) in the indicated groups. Scale bar, 3 mm. L) Masson's trichrome staining images of gastrocnemius muscles in the indicated groups. The enlarged regions are boxed within yellow dotted lines. Scale bars, 200 µm for upper panels, 20 µm for lower panels. M–O) Quantitative analysis of muscle weight recovery ratio (M), gastrocnemius muscle fiber diameters (N), and collagen area proportions (O). Six rats in the SC and ASC groups and five rats in the autograft and eton groups. P) Comparison of functional recovery in rat PNI models treated with autograft, eton, SC, and ASC for 14 weeks. Data were presented as mean ± SD; *, *P* < 0.05; **, *P* < 0.01; ***, *P* < 0.001; N.S., not significant; ANOVA.

The above results revealed that ASC could promote the structural regeneration and remyelination of damaged nerves, which serve as the basis for transmitting signals to target muscles for executing functions. To evaluate functional recovery, paw withdrawal latency, hind limb grip force, and sciatic function index (SFI) were assessed at various time points post‐transplantation^[^ ^100^, ^101^, ^102^, ^103^, ^104^ ^]^ (**Figure** **7**A). Rat weights increased gradually across all groups post‐transplantation, with no significant differences (Figure 7B). Paw withdrawal latency significantly increased after surgery, gradually decreasing over time. The ASC group exhibited significantly lower latency compared to the eton and SC groups, while showing no significant difference from the autograft group (Figure 7C). The hind limb grip force significantly decreased post‐transplantation and gradually recovered. The ASC group exhibited grip force restoration comparable to the autograft group and significantly higher than the eton and SC groups (Figure 7D). Fourteen weeks post‐transplantation, the ASC group showed an SFI comparable to the autograft group and significantly lower than the eton and SC groups (Figure 7E,F). Taken together, these results indicate that ASC could achieve autograft‐comparable behavioral recovery in long‐gap limiting size PNI repair. Notable behavioral recovery was observed in both the ASC and autograft groups, although it failed to reach the normal levels, likely due to target organ atrophy caused by prolonged denervation.^[^ ^105^, ^106^ ^]^

To asses signal transmission, an electrophysiological examination was performed 14 weeks post‐transplantation (Figure 7G). ASC effectively promoted the restoration of nerve conductivity (Figure 7H). The ASC group demonstrated significantly higher nerve conduction velocity (NCV) and compound muscle action potential (CMAP) than the SC group. Moreover, the ASC group showed 1.6‐fold and 5.2‐fold higher NCV and CMAP values than the eton group, respectively (Figure 7I,J).

To detect the restoration of target muscles innervated by the sciatic nerve, the gastrocnemius muscles were collected 14 weeks post‐transplantation and subjected to Masson's trichrome staining (Figure 7K,L). A minimal amount of collagen was observed in the ASC and autograft groups. Conversely, an enormous amount of collagen was deposited around the muscle fibers in the eton and SC groups. Muscle weight analysis revealed that gastrocnemius muscle atrophy was significantly alleviated in the ASC and autograft groups (Figure 7M). The ASC group exhibited a significantly larger gastrocnemius muscle fiber diameter compared to the SC groups, which measured 1.4‐fold larger than the eton group. Additionally, the collagen fiber area in the ASC group was comparable to that of the autograft and markedly lower than in the SC group, showing a 75% reduction relative to the eton group (Figure 7N,O). This favorable outcome may result from the rapid reconstruction of innervating nerves, along with the alleviating effect of silicates on muscle atrophy.^[^ ^107^ ^]^ Taken together, a comparative analysis of functional restoration demonstrated that ASC outperformed the commercial eton conduits and achieved behavioral recovery comparable to autografts, the clinical gold standard (Figure 7P). The ASC group remained slightly inferior to the autograft group in certain indices, likely because peripheral nerve regeneration is a complex pathophysiological process involving molecular, cellular, and tissue changes, and the regenerative process cannot be fully achieved by regulating Schwann cells alone. To further enhance outcomes in long‐gap peripheral nerve regeneration, additional strategies could be combined, such as using 4D printing techniques to construct well‐ordered topological microstructures, combining ultrasound or magnetic fields to modulate electrical stimulation and drug release, or introducing novel materials to optimize the mechanical stimulation of conduit.

### 2.8 ASC Possesses Favorable Barrier Ability, Biodegradability, and Biosafety

**Figure 8:**
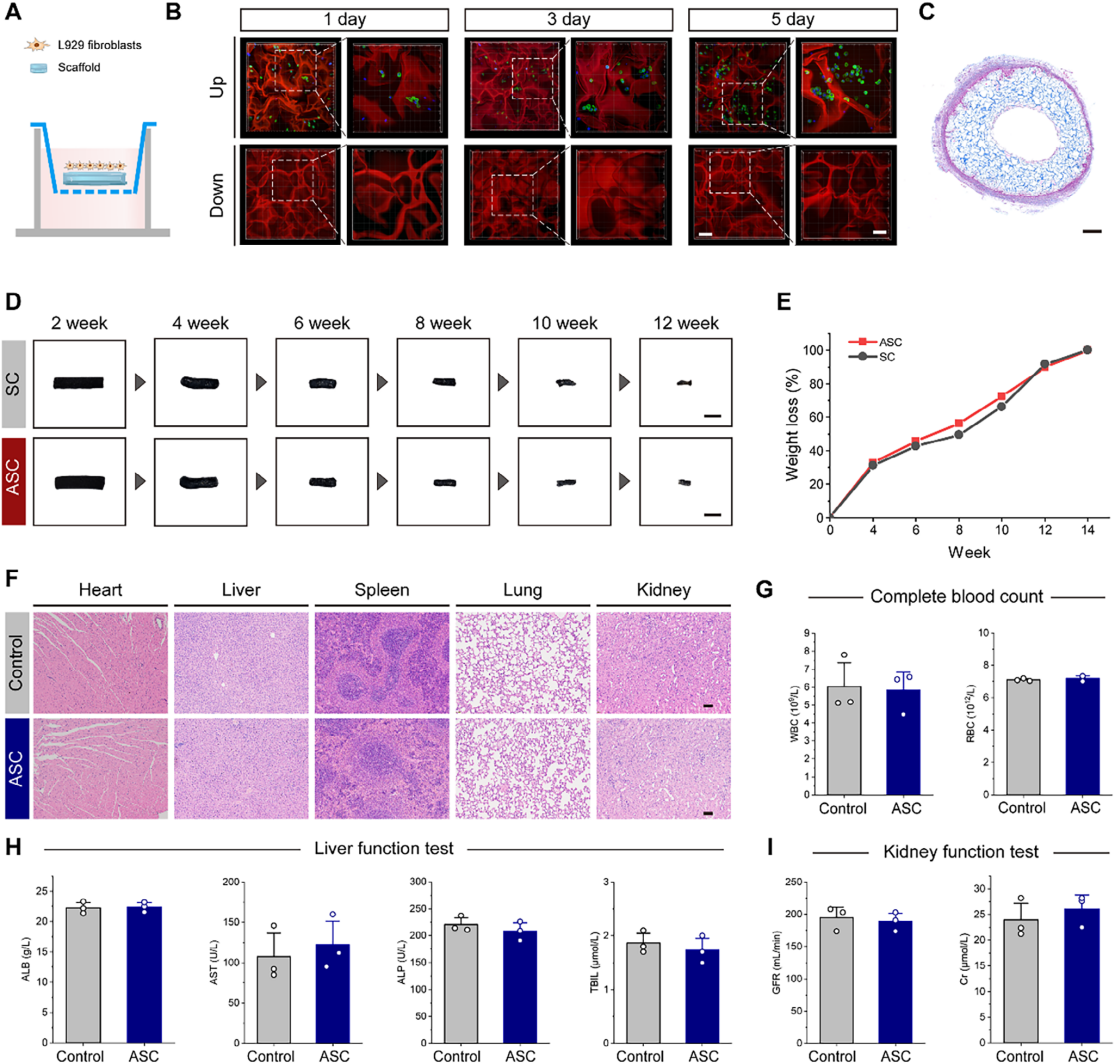
Cell barrier ability, degradation, and biosafety of nerve conduits. A) Illustration of L929 fibroblasts cultured on the AT‐SS scaffold. B) Phalloidin staining of L929 fibroblasts growing on the AT‐SS scaffold for 5 days. Red indicates the self‐fluorescence of the scaffold, and green represents phalloidin‐stained fibroblasts. Scale bars: 100 µm for left panel, and 50 µm for right panel. C) Masson's trichrome staining of ASC 15 days after transplantation. Scale bar, 500 µm. D,E) Macroscopic views of SC and ASC at various time points following in vivo transplantation are shown in (D), and the weight loss was measured and calculated (E). Scale bar, 5 mm. F) H&E staining of heart, liver, spleen, lung, and kidney tissue specimens of rats 2 weeks after ASC transplantation. Scale bar, 100 µm. G–I) Complete blood count (G), liver function test (H), and kidney function test (I) 2 weeks after ASC transplantation (*n* = 3). Data were presented as mean ± SD; ANOVA.

Excessive fibrous tissue invading the interior of the conduit can impede nerve regeneration.^[^ ^108^ ^]^ To evaluate fibrous tissue invasion, L929 fibroblasts were cultured on the upper layer of AT‐SS scaffold for 5 days. Phalloidin staining showed that the cells remained confined to the upper layer, with no cells detected in the lower layer (**Figure** **8**A,B). Fifteen days after in vivo transplantation, Masson's trichrome staining revealed that collagen fibers were predominantly localized around the ASC, with no significant internal invasion observed (Figure 8C). These results confirmed the conduit's barrier ability to prevent fibrous tissue infiltration. Regarding in vivo degradation, both SC and ASC degraded gradually after transplantation (Figure 8D), reaching ≈90% degradation by week 12 and complete degradation by week 14 post‐implantation (Figure 8E). This slow degradation of ASC suggests its ability to persistently enhance the pro‐regenerative capacity of Schwann cells, thereby providing a sustained therapeutic advantage for long‐gap PNI regeneration. H&E staining of the heart, liver, spleen, lung, and kidney tissue specimens 2 weeks post‐implantation indicated no significant organ damage (Figure 8F). Additionally, complete blood count, liver and kidney function tests showed no abnormalities (Figure 8G–I), confirming the excellent biosafety of ASC and highlighting its potential for clinical translation.

## 3 Conclusion

In the current study, we systematically evaluated the bioactivities of six bioceramics and found that bioceramic AT exhibited superior cytocompatibility, neurotrophic factor secretion, and anti‐inflammatory capacity. Further assessments revealed that AT substantially enhanced the proliferation, migration, and secretion functions of Schwann cells by activating the PI3K/AKT and MAPK/ERK signaling pathways. Given that silk sericin possesses neurotrophic and neuroprotective bioactivities, and contains an abundance of functional groups for further modification, we chelated SS with AT to fabricate a composite nerve conduit for repairing a long‐gap limiting size PNI. Given the distinctive acidic amino acid composition of SS, which facilitated Ca and Mg release from AT, the combination of AT with SS synergistically enhanced the pro‐regenerative function of Schwann cells. The developed AT‐SS composite conduit possessed optimal physical characteristics while simultaneously improving Schwann cell function in vivo and accelerating axonal elongation. In a 13‐mm limiting size PNI model, the AT‐SS conduit achieved efficient nerve regeneration, matching autograft performance in key functional indices, and outperformed the commercial eton conduit across multiple parameters. The composite conduit exhibited excellent degradation and biosafety. Collectively, these findings establish an innovative ion‐based therapeutic approach to repair long‐gap limiting size PNI using an innovative bioceramic/SS composite conduit.

## 4 Experimental Section

### Preparation of the Bioceramics Dissolution Extract

Bioceramics, including CS, Cu‐CS, HT, DI, BT, and AT, were acquired from the State Key Laboratory of High Performance Ceramics and Superfine Microstructure, Shanghai Institute of Ceramics, Chinese Academy of Sciences. Original dissolution extracts of bioceramics were prepared according to the International Standard Organization (ISO/EN 10993–5).^[^ ^109^ ^]^ Briefly, 200 mg of bioceramics powders were added to 1 mL of Dulbecco's modified Eagle's medium (DMEM, Hyclone, USA) for incubation at 37 °C. These mixtures were shaken at 120 rpm for 24 h, and the suspension was centrifuged at 4000 rpm for 10 min. The original dissolution extracts of bioceramics were collected and sterilized with a 0.22 µm filter. The diluted AT dissolution extract was prepared by adding fresh DMEM at ratios of 1/32 and 1/64, along with 1% penicillin/streptomycin (Hyclone, USA).

### SS Extraction and Fabrication of Scaffolds and Conduits

SS was extracted from the cocoons of fibroin‐deficient silkworm strains (140 Nd‐s *Bombyx mori*) using the LiBr method.^[^ ^43^ ^]^ Briefly, cocoon pieces were dissolved in 1 m LiBr at 35 °C for 24 h. After dialysis with distilled water and concentration with PEG‐6000 solution, 10 mg mL^−1^ of sericin solution was detected using the BCA method (BL521A; Biosharp, China). Based on the authors’ previous studies on the relationship between sericin/genipin ratios and mechanical properties,^[^ ^43^, ^110^ ^]^ 6 mL SS was mixed with 1 mL genipin (1%, w/v, dissolved with distilled water), followed by the dispersion of 20 mg AT with 1 mL of mixture to prepare precursor solution, which was added to the bottom of 24‐well plates or into conduit molds at 37 °C for 24 h to form hydrogels. After being frozen at −80 °C and lyophilization, SS‐based scaffolds and nerve conduits were constructed and then sterilized with 75% ethanol for further experiments.

### Cytocompatibility and Cell Proliferation Analysis

For evaluating the cytocompatibility of bioceramics, RSC 96 cells seeded in 96‐ or 12‐well plates were cultured with the original dissolution extracts of different bioceramics at 37 °C for 24 h. Cell viability and survival were tested with CCK‐8 (Dojindo, Japan) and Live and Dead staining assay (Biovision, USA) according to their manufacturers’ instructions. To test the effects of AT on Schwann cell proliferation, RSC 96 cells seeded in 96‐ or 12‐well plates were incubated with 1/32 and 1/64 diluted dissolution extracts of AT, along with 10% fetal bovine serum (FBS; Gibco, USA). After incubation for 48 h, cell viability was evaluated using the CCK‐8 assay, while cell proliferation was detected by performing anti‐Ki67 (28074‐1‐AP; Proteintech, China) immunofluorescence staining. Coralite488‐conjugated goat anti‐rabbit IgG (SA00013‐2; Proteintech, China) was used as the secondary antibody.

### Hemolysis Assay

Hemolysis was examined as described previously.^[^ ^111^ ^]^ Briefly, a 2% erythrocyte suspension was prepared by diluting murine blood with PBS. And 1 mg of bioceramic powder was added to 200 µL of suspension. Following incubation at 37 °C and shaking at 100 rpm for 2 h, the supernatant was collected after centrifugation for 10 min at 8000 rpm. A microplate reader was used to detect the specific absorbance of hemoglobin at 545 nm. The hemolysis ratio was calculated using the following equation

(1) $$\mathrm{Hemolysis}\;\left(\%\right)=\frac{O.D._{\mathrm{sample}}-\;O.D._{\mathrm{negative}}}{O.D._{\mathrm{positive}}-\;O.D._{\mathrm{negative}}}\times 100\%$$

where O.D._positive_, O.D._negative_, and O.D._sample_ represent the absorbance values of Triton X‐100, PBS, and bioceramic groups, respectively.

### Scratch Test

After RSC 96 cells were cultured as a monolayer on 48‐well plates, a 200 µL sterile pipette tip was used to create a linear wound at the midline. Subsequently, the remaining cell debris was removed using PBS, and cells were incubated with diluted dissolution extract of AT for 5 days; micrographs of the cells were recorded daily. The wound healing rate was analyzed using ImageJ software (1.48v, NIH, USA).

### Transwell Test

Upon seeding RSC 96 cells on the Boyden chambers (8 µm, 24‐well plates, Corning, USA), 200 µL FBS‐free DMEM was added to the upper chamber, while 500 µL diluted dissolution extract of AT was added to the lower chamber. After a 20‐h incubation, the cells were fixed with 4% paraformaldehyde and stained with 0.5% crystal violet. After removing the cells from the inner surface, the migrated cells on the outer surface were photographed with a microscope (IX 71; Olympus, Japan). To detect the effect of AT‐SS conduit on RSC 96 cell migration, the AT‐SS scaffolds were placed in the lower chamber. RSC 96 cells were seeded in the upper chamber and incubated at 37 °C for 20 h. Migrated cells were detected as described above and analyzed with ImageJ software.

### Quantitative Real‐Time PCR

To evaluate the cell secretion, RSC 96 cells were cultured with dissolution extracts of bioceramics or scaffolds for 24 h. mRNA was isolated using TRIzol reagents (Invitrogen, Carlsbad, CA, USA) and transcribed into cDNA. The desired mRNA transcript was detected using AceQ Universal SYBR qPCR Master Mix (Vazyme, China) according to a previously described method.^[^ ^43^, ^112^ ^]^ To assess the impacts of bioceramics on inflammation, RAW 264.7 cells were first stimulated with 1 µg mL^−1^ of LPS (L2630, Sigma, USA) for 2 h, followed by an 8‐h co‐incubation with LPS and various undiluted bioceramic extracts. The mRNA was isolated and analyzed as described above. The primers used are listed in the Supporting Information (Table S1, Supporting Information).

### Western Blotting Test

RSC 96 cells were cultured with a diluted dissolution extract of AT for 12 h and subjected to western blotting tests as described previously.^[^ ^42^, ^113^ ^]^ Cellular proteins were isolated and detected using BCA assay reagents. After SDS‐PAGE electrophoresis, proteins were transferred to nitrocellulose membranes, antibodies: anti‐PI3K (20584‐1‐AP; Proteintech, China), anti‐AKT (10176‐2‐AP; Proteintech, China), anti‐p‐AKT (Ser473; 4060; CST, USA), anti‐mTOR (2983; CST, USA), anti‐p‐mTOR (Ser2448; 67778‐1‐lg; Proteintech, China), anti‐MEK1/2 (11049‐1‐AP; Proteintech, China), anti‐p‐MEK1/2 (Ser217/221; 9154; CST, USA), anti‐ERK1/2 (p44/42; 4695; CST, USA), anti‐p‐ERK1/2 (Thr202/Tyr204; 4370; CST, USA), and anti‐β‐actin (81115‐1‐RR; Proteintech, China) were incubated. A luminescent image analyzer (Fujifilm LAS‐4000, Japan) was used to examine the blots, which were analyzed using the ImageJ software.

### Transcriptomic Analysis

For RNA sequencing analysis, RSC 96 cells were cultured with 1/64 diluted dissolution extract of AT in 6‐well plates for 24 h, and their total RNA was extracted with TRIzol reagents. RNA sequencing was serviced by Novogene Co., Ltd. (China). Raw read counts were normalized using DESeq2. Differential expression analysis was conducted using a threshold of *P*‐value ≤0.05 and |log2foldchange| ≥1. GO analysis was performed using a significance threshold of *P*‐value <0.05. GSEA was conducted using the BIOCARTA_AKT_PATHWAY and BIOCARTA_ERK_PATHWAY gene sets to evaluate pathway‐level changes.

### Characterization of Conduits

The porous microarchitectures of the conduits were detected using a scanning electron microscope (ULTRA PLUS‐43‐13; Zeiss, Germany). The porosities of conduits were analyzed using the ethanol displacement method,^[^ ^114^ ^]^ and calculated using the following equation

(2) $$\mathrm{Porosity}\;\left(\%\right)=\frac{W_{i}-W_{0}}{V\;\times \rho }\times 100\%$$

where *W*_0_ and *V* were initial dried mass and volume, *W*_i_ was the mass after immersion, *ρ* is 0.789 g cm^−3^, the density of ethanol.

The permeability was measured as reported previously.^[^ ^84^ ^]^ A Franz diffusion cell with a 9 mm orifice, 4.5 mL receptor volume was used. The donor chamber contained 0.6 mL of TB solution (9 mg mL^−1^), while the receptor chamber contained PBS maintained at 37 °C with constant stirring at 1000 rpm. ASC was cut into membranes and placed between the chambers, and 50 µL samples were collected every 20 min for concentration analysis.

Swelling behavior was detected as described previously.^[^ ^45^ ^]^ Following lyophilization, the conduits were soaked in PBS at 37 °C for 7 days. The conduit weight was measured, and the swelling ratio was calculated as follows

(3) $$\mathrm{Swelling}\;\left(\%\right)=\frac{W_{t}\;-\;W_{0}}{W_{0}}\times 100\%$$

where *W*_0_ represents the initial dry weight of conduit, and *W*_t_ is the swollen mass at different time points.

The tensile modulus and compressive modulus were examined using a universal testing machine (Instron 5848 MicroTester; USA) at a speed of 1 mm min^−1^ at room temperature. After grinding the lyophilized conduits into powders, the secondary structures were detected using an FTIR spectroscope (Nicolet 6700; Thermo, USA) and analyzed using the OMNIC software.

### Ion Detection

To analyze ion release, 20 mg of AT or ASC (containing 20 mg AT) was incubated in 1 mL of deionized water at 37 °C with shaking at 120 rpm. The supernatant was collected after centrifugation at 12 000 rpm for 20 min. The Ca, Mg, and Si concentrations were measured using an atomic absorption spectrometer (Z‐2300; Hitachi, Japan). To evaluate intracellular ions, RSC 96 cells were treated with 1/64 or 1/32 diluted AT extracts for 24 h. After washing with PBS for 5 times, 2 × 10^6^ cells per sample were resuspended in deionized water and sonicated for 5 min. The supernatant was collected after centrifugation at 12 000 rpm for 20 min, and atomic absorption spectrometry was performed to detect Ca, Mg, and Si concentrations. Additionally, intracellular Mg and Ca levels were monitored using probed ethyl benzo[6,7]‐4‐*oxo*‐4H‐quinolizine‐3‐carboxylate (E332021; Aladdin, China) and Fura‐2 AM (S1052; Beyotime, China) at a concentration of 2 µm, respectively.

### Detection of Conduit Barrier Ability

For in vitro evaluation, AT‐SS scaffolds were added to Boyden chambers (8 µm, 24‐well plates, Corning, USA) and cultured with L929 cells for 5 days. Samples were fixed with 4% paraformaldehyde and stained with phalloidin at the indicated time points. Images of the upper and lower layers of the scaffolds were obtained. For in vivo evaluation, ASC was transplanted into rats for 15 days. Cross‐sections of the middle sites of conduits were prepared and subjected to Masson's trichrome staining.

### Long‐Gap Sciatic Nerve Transection Model and Treatment

All surgical procedures were approved by the Animal Care and Use Committee, Tongji Medical College, Huazhong University of Science and Technology, Wuhan, China (The ethical approval number: [2023] IACUC No. 3765). Commercial nerve conduits were purchased from Yitong Bio‐technology Co., Ltd. (Jiangsu, China). Sprague–Dawley rats (250–300 g) were anesthetized, and the right hind limb was shaved and sterilized. The skin and muscles were excised to expose the sciatic nerve. A 13‐mm nerve segment was cut to establish a long‐gap limiting size sciatic nerve defect model. After placing 1 mm nerve stumps into the lumen of conduit, a 15‐mm conduit was sutured to the defect with 8‐0 nylon sutures. In autograft group, the removed nerve segment was reverse‐sutured to the stumps. The wounds were closed and sterilized with iodine. Within 24 h post‐operation, one rat from the autologous group and one from the eton group died due to undetermined causes. Consequently, these data were excluded from the analysis. Finally, the SC and ASC groups comprised six rats each, respectively, while the autograft and eton groups comprised five rats each.

### Isolation of Primary Schwann Cells

To purify the primary Schwann cells,^[^ ^115^, ^116^ ^]^ the conduits were removed and cut into pieces on ice 20 days post‐transplantation. The fragments were digested with 10 mL of composite enzyme solution containing 5 mg mL^−1^ collagenase and 1 mg mL^−1^ trypsin at 37 °C for 1 h. After adding 200 µL FBS to terminate the digestion, the suspensions were filtered through a 40‐µm cell strainer. Supernatants of the suspensions were discarded after centrifugation at 1000 rpm for 5 min. The cell pellets were resuspended in DMEM and incubated with anti‐P75^NTR^ antibody (55014‐1‐AP; Proteintech, China) and FITC‐conjugated goat anti‐rabbit IgG (SA00003‐2; Proteintech, China) at 4 °C for 2 h. The primary Schwann cells were collected by sorting the positive P75^NTR^ cells with fluorescence‐activated cell sorting. For identification, the collected cells were fixed with 4% paraformaldehyde for 5 min and stained with anti‐S100 antibody (ab4066; Abcam, U.K.) and Coralite647‐conjugated goat anti‐mouse IgG (RGAM005; Proteintech, China). Flow cytometry was performed to determine the proportion of P75^NTR^ and S100 double‐positive cells.

### Histological Assessments

The implanted conduits or regenerated nerves were excised from the rat models 15 days or 14 weeks post‐surgery, fixed in 4% paraformaldehyde for 24 h, and embedded in paraffin. After cutting the conduits or regenerated nerves into 4‐µm–thick sections, the Schwann cells and axons were visualized by immunofluorescence staining with the anti‐S100 antibodies and anti‐Tuj‐1 (ab52623; Abcam, U.K.). The secondary antibodies used were as follows: Coralite488‐conjugated goat anti‐rabbit IgG (RGAR002; Proteintech, China), FITC‐conjugated goat anti‐mouse IgG (SA00003‐1; Proteintech, China), and CY3‐conjugated goat anti‐rabbit IgG (SA00009‐2; Proteintech, China). Nuclei were stained with 4′,6‐diamidino‐2‐phenylindole (DAPI; Boster, China). The images were acquired by an inverted fluorescence microscope and analyzed using ImageJ.

### Morphometric Analysis of Myelin

Fourteen weeks post‐surgery, the regenerated nerves were collected and fixed with 2.5% glutaraldehyde at 4 °C for 24 h. After embedding in resin, semi‐thin sections of the middle and distal sites were prepared and stained with TB. Ultrathin cross‐sections at the distal sites of regenerated nerves were prepared and detected using a transmission electron microscope (JEM‐1400plus; Japan). The myelin thickness, diameter of myelinated nerve fibers, and diameter of inner axon were measured with ImageJ software. The g‐ratio was calculated as the diameter of the axon divided by the diameter of nerve fibers.

### Behavioral Tests

Fourteen weeks post‐surgery, the behavioral restoration was evaluated as described previously.^[^ ^100^, ^101^, ^102^, ^103^, ^104^ ^]^ Paw withdrawal latency was evaluated using Hargreaves tests (PL201; Techman, China), and hind limb grip force was measured using a grip strength meter (ZP‐5; AIGU, China). Baseline values were assessed 2 weeks pre‐operation. After the rats walked through a confined walkway (10 cm wide × 10 cm high × 60 cm long), the inked white paper of the hind‐paw footprints was scanned using a scanner (LIDE300; Canon, Japan). The toe spread, print length, and intermediary toe spread of both the experimental and normal hind paws were determined using ImageJ. The SFI was calculated as follows

(4) $$\begin{array}{c} \mathrm{SFI}\;=-38.3\times \frac{PL_{E}-\;PL_{N}}{PL_{N}}+109.5\times \frac{TS_{E}-\;TS_{N}}{\;TS_{N}}\times \frac{IT_{E}-\;IT_{N}}{IT_{N}}-8.8 \end{array}$$

where TS represents the distance from the first toe to the fifth toe, PL indicates the distance from the heel to the third toe, IT is the distance between the middle of the second and fourth toes, and the subscripts E and N denote the experimental and normal paws, respectively.

### Electrophysiologic Examination

Fourteen weeks post‐surgery, nerve conduction and muscle activity were evaluated using electromyography as reported previously.^[^ ^117^ ^]^ Following anesthesia induction, the regenerated sciatic nerve and gastrocnemius muscle were exposed. An electrophysiological recording system (BL‐420L; TECHMAN, China) was used to apply electrical stimuli separately at the proximal and distal ends of the regenerated nerve, while the recording electrodes were placed on the gastrocnemius muscle. The CMAP was recorded, and NCV was calculated using the latency and distance between the two nerve stumps.

### Gastrocnemius Muscle Evaluation

The operational gastrocnemius muscles of treated rats were harvested and weighed 14 weeks post‐transplantation. Following fixation with 4% paraformaldehyde, Masson's trichrome staining was performed on cross‐sections of the muscles as described previously.^[^ ^45^ ^]^ Images were collected using a microscope, and the muscle fibers and collagen were analyzed using the ImageJ software.

### Evaluation of Conduit Degradation and Biosafety

To assess degradation, 15‐mm–long conduits were implanted into rats and retrieved at indicated time points for macroscopic observation. The lyophilized weights were recorded to determine residual mass over time. For biosafety evaluation, a conduit was implanted into rats. Animals were sacrificed 2 weeks later. And major organs (heart, liver, spleen, lungs, and kidneys) were harvested and subjected to H&E staining; blood samples were collected for complete blood count and liver/kindey function examination.

### Statistical Analysis

Data were analyzed with OriginPro 2021 and presented as mean ± SD. Data normality was assessed using the Shapiro–Wilk test, with all datasets showing *P* > 0.05, confirming a normal distribution. Variance homogeneity was evaluated using Levene's test. Student's *t*‐tests were used to compare two‐groups, while one‐way ANOVA was used for multi‐group comparisons. Post hoc analysis was performed using Scheffe's test for unequal sample sizes and Tukey's test for equal sample sizes.

## Conflict of Interest

The authors declare no conflict of interest.

## Supporting information



Supporting Information

## Data Availability

The data that support the findings of this study are available from the corresponding author upon reasonable request.
