# Supplementary material for: Bioactive Silk Sericin/Bioceramic Nerve Guidance Conduit for Effective Repair of Long‐Gap Transected Peripheral Nerve Injury through Regulating Schwann Cells
Source: Adv Sci (Weinh). 2025 Jul 8;12(38):e07241. doi: 10.1002/advs.202507241 (PMC12520579; doi:10.1002/advs.202507241)
Supplement: Supplementary file 1 — Supporting Information [file ADVS-12-e07241-s001.docx]

Supporting Information

Bioactive Silk Sericin/Bioceramic Nerve Guidance Conduit for Effective Repair of Long-Gap Transected Peripheral Nerve Injury through Regulating Schwann Cells

*Qiangfei Su, Jian Wang, Yu Song,* *Zhaowenbin Zhang, Bo Cai, Xiakeerzhati Xiaohalati, Jingwei Liu, Haozhe Li, Zheng Wang*, Jiang Chang*, and Lin Wang**


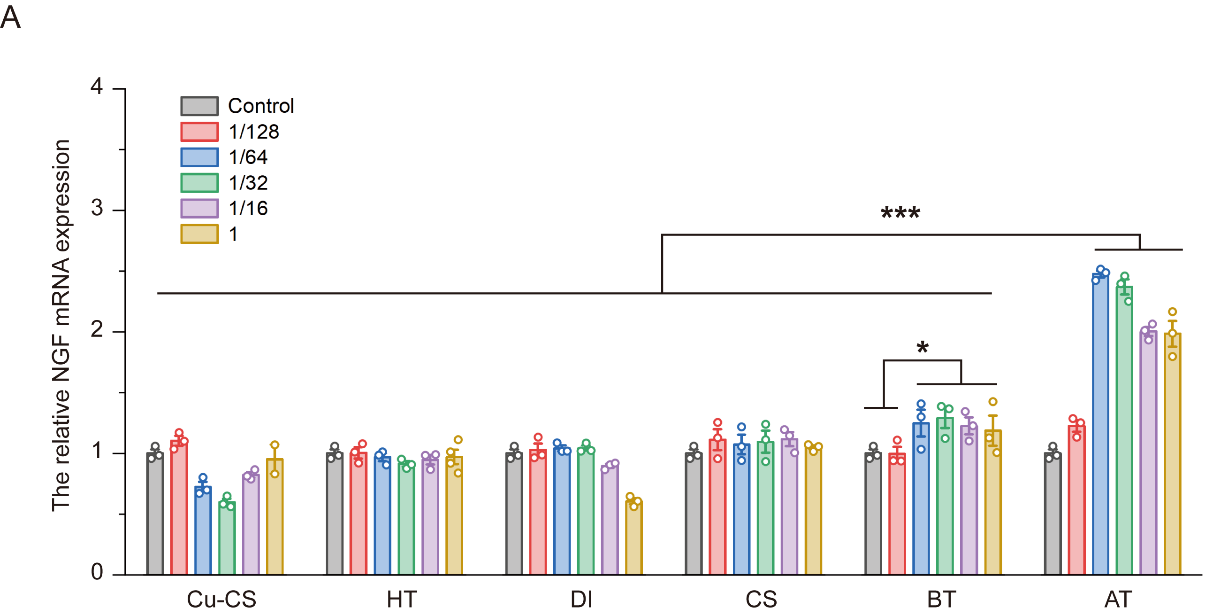


**Figure S1.** The relative mRNA expression of NGF in RSC 96 cells treated with diluted extracts of various bioceramics (*n* = 3). Data were presented as mean ± SD; *, *P* < 0.05; ***, *P* < 0.001; ANOVA.


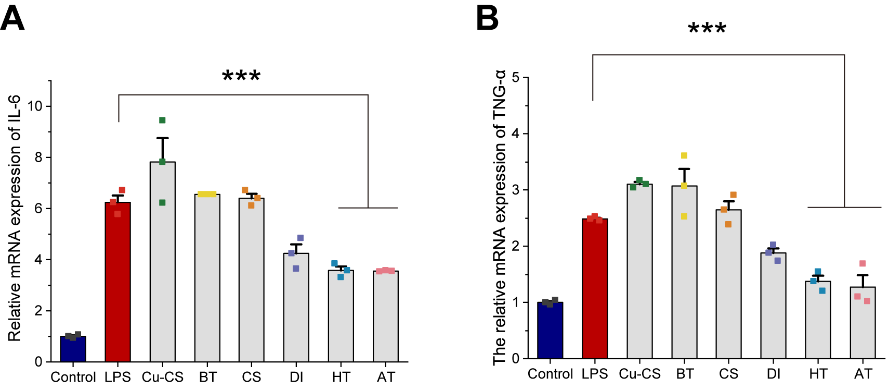


**Figure S2.** Relative mRNA expression of IL-6 and TNF-α in LPS-stimulated RAW 264.7 cells treated with original dissolution extracts of different bioceramics (*n* = 3). Data were presented as mean ± SD; ***, *P* < 0.001; ANOVA.


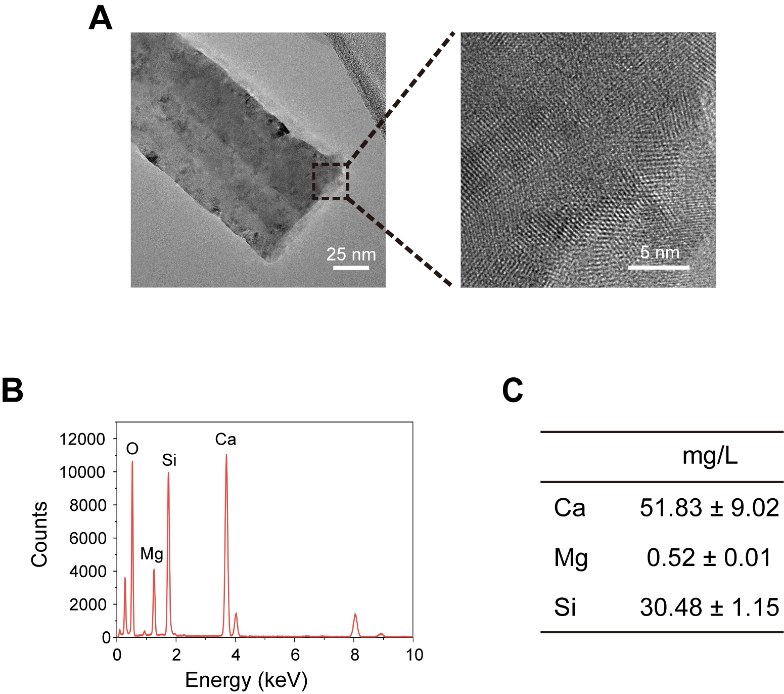


**Figure S3.** Characterization of AT and elemental content in its extract. (A) High-resolution transmission electron microscopy images of AT. The enlarged region was boxed with black dashed lines. Scale bars, 25 nm for left panels, 5 nm for right panels. (B) EDS spectrum results of AT. (C) Elemental absorption spectroscopy results of Ca, Mg, and Si ions in AT extract (*n* = 3). Data were presented as mean ± SD.


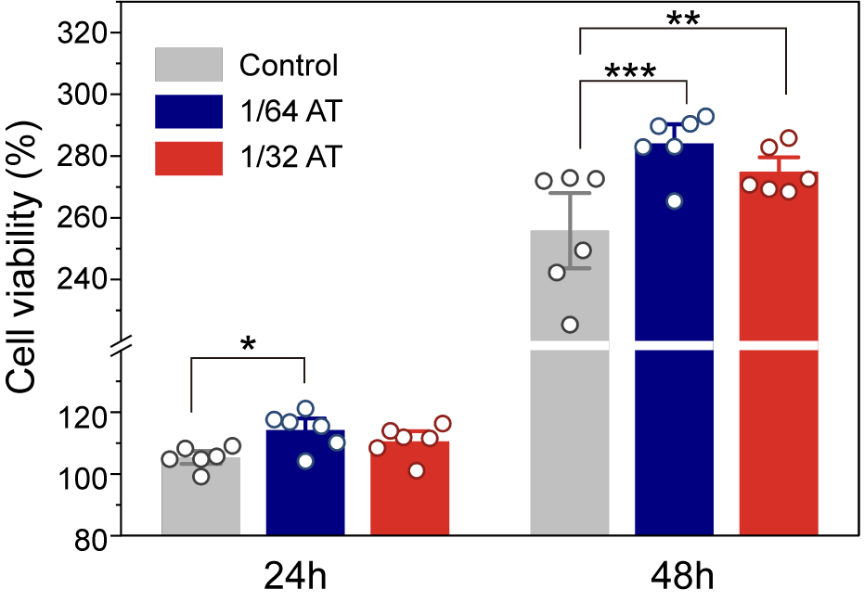


**Figure S4.** The cell viability of RSC 96 cells treated with diluted AT dissolution extracts for 24 or 48 hours (*n* = 6). Data were presented as mean ± SD; *, *P* < 0.05; **, *P* < 0.01; ***, *P* < 0.001; ANOVA.


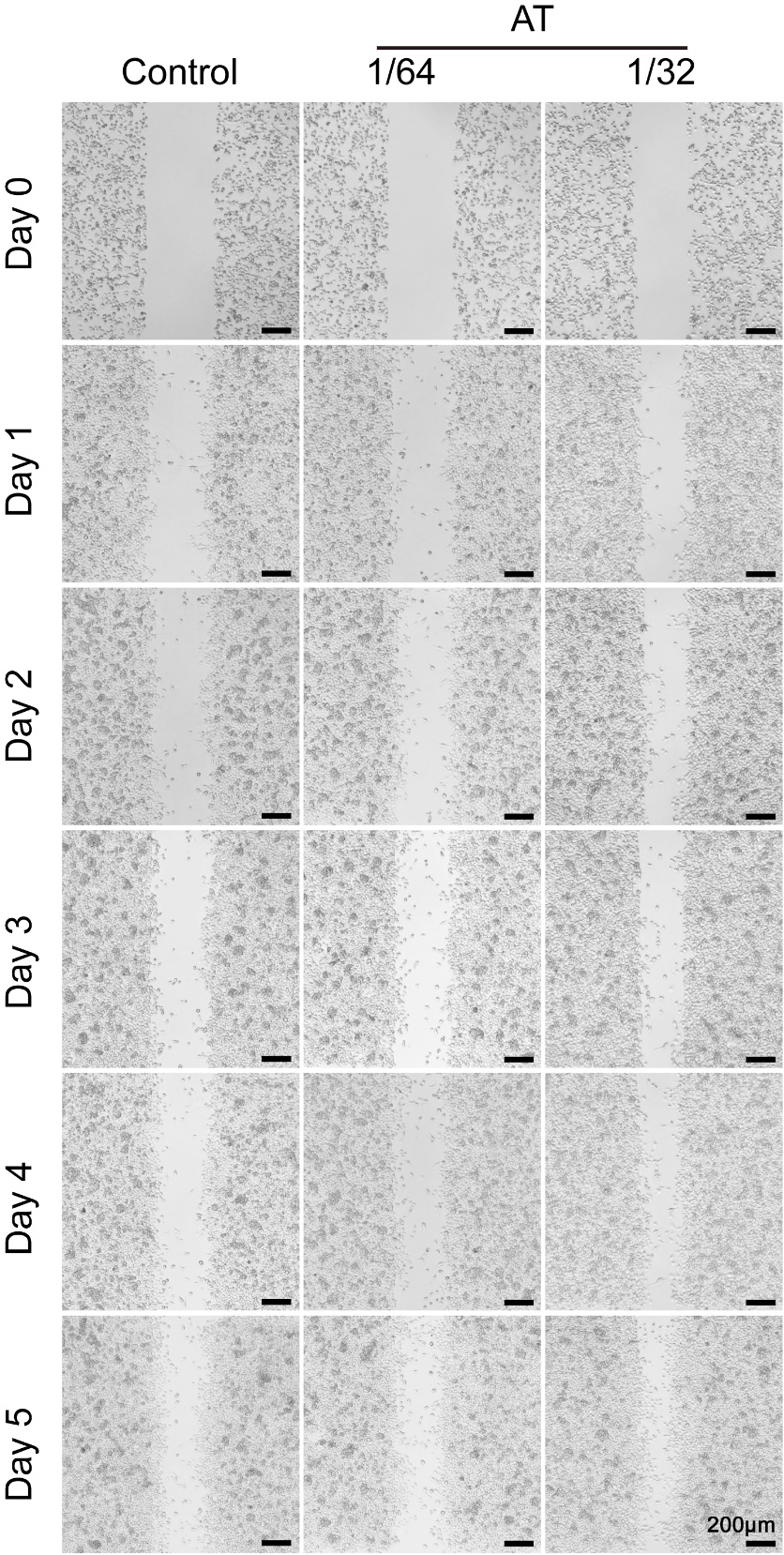


**Figure S5.** Wound healing images of RSC 96 cells cultured with diluted AT dissolution extracts for 5 days in Scratch test. Scale bar, 200 μm.


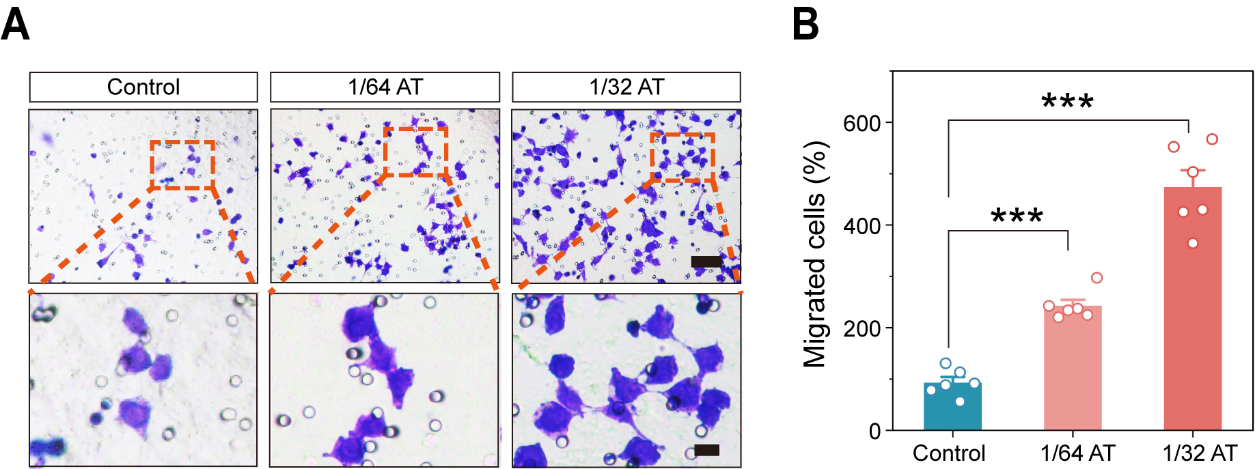


**Figure S6.** Transwell test for RSC 96 cells cultured with diluted AT dissolution extracts for 20 hours. (A) Crystal violet staining images of RSC 96 cells. Enlarged regions are boxed with red dashed lines. Scale bars, 200 μm for upper panels, 50 μm for lower panels. (B) The proportion of migrated cells (*n* = 6). Data were presented as mean ± SD; ***, *P* < 0.001; ANOVA.


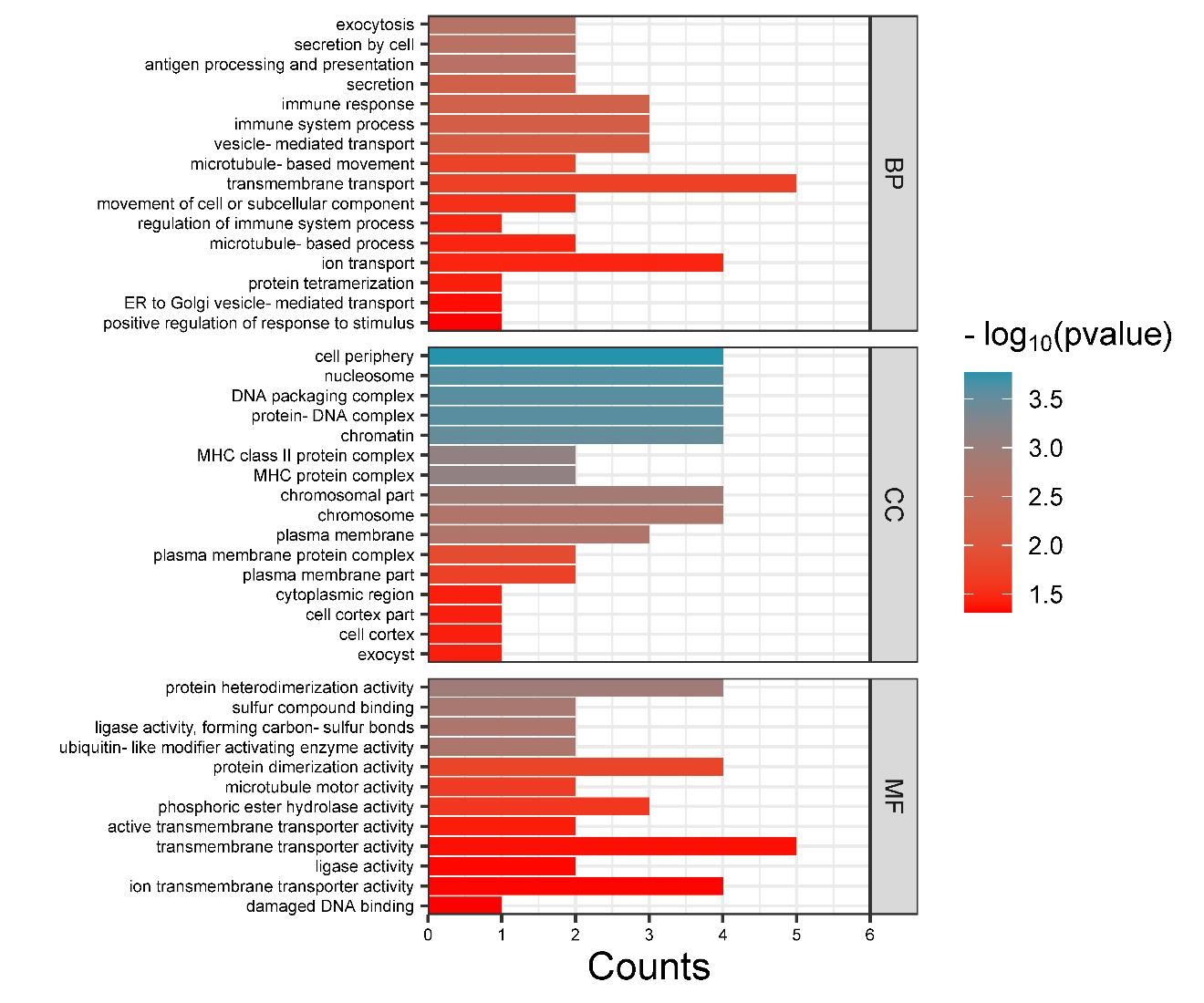


**Figure S7.** GO enrich bar graphs of DEGs. BP, biological process. CC, cell component. MF, molecular function.


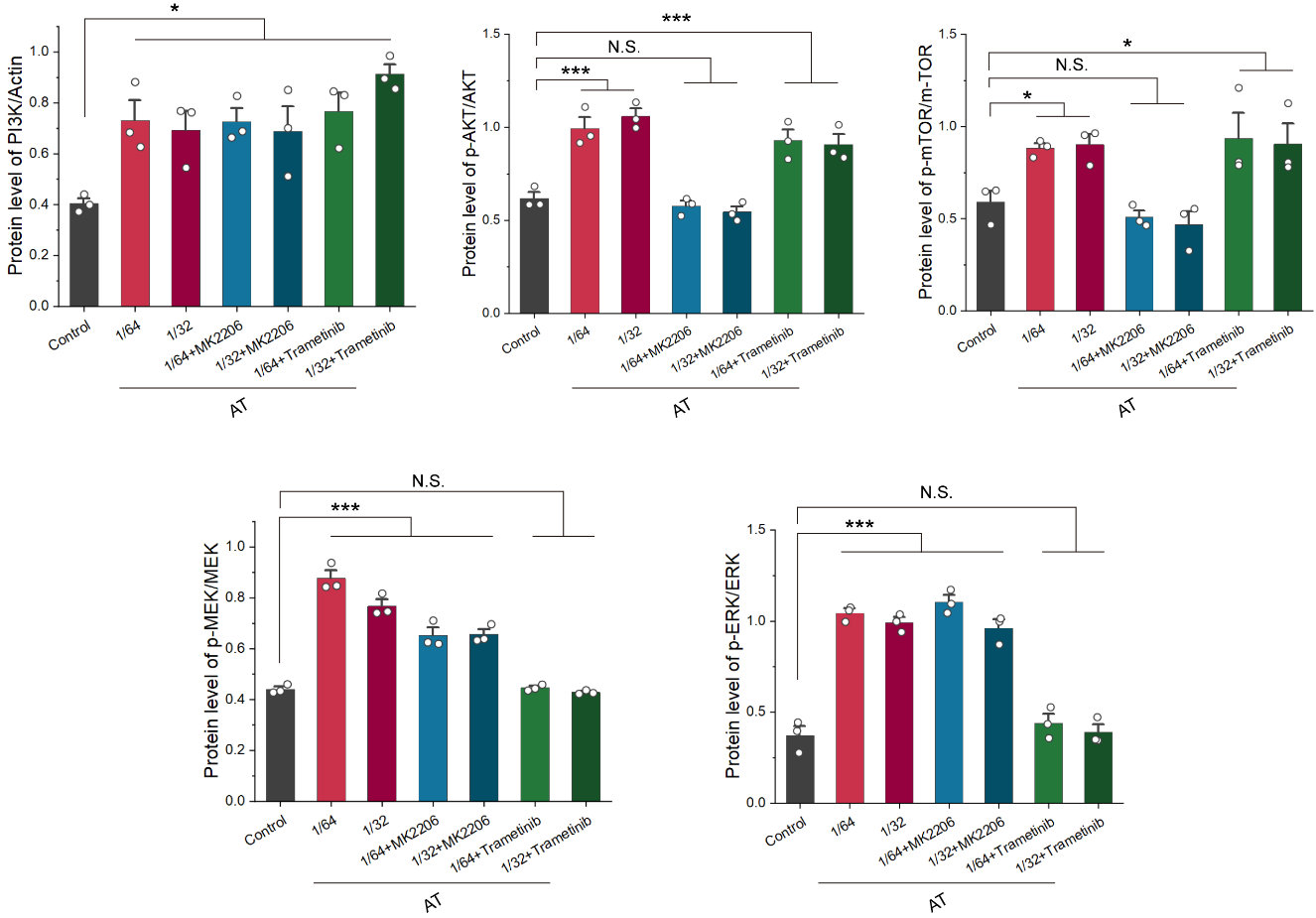


**Figure S8.** Relative protein levels of PI3K, p-AKT, p-mTOR, p-MEK, and p-ERK in RSC 96 cells treated with diluted AT extracts upon the existence of AKT inhibitor (MK2206) and the MEK inhibitor (Trametinib) or not (*n* = 3). Data were presented as mean ± SD; *, *P* < 0.05; ***, *P* < 0.001; N.S., not significant; ANOVA.


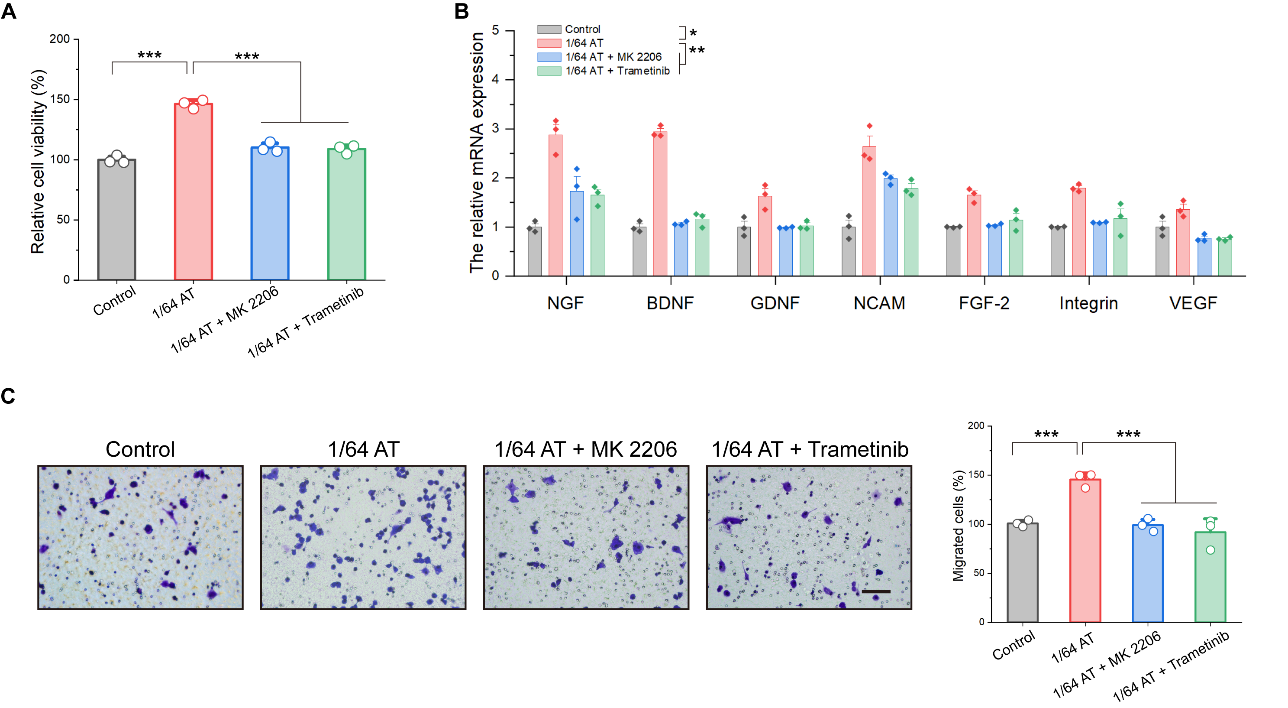


**Figure S9.** The relative cell viability (A), mRNA expression (B), and cell migration (C) of RSC 96 cells treated with AKT inhibitor (MK2206) or MEK inhibitor (Trametinib), respectively. (*n* = 3). Scale bar in (C), 100 μm. Data were presented as mean ± SD; *, *P* < 0.05; **, *P* < 0.01; ***, *P* < 0.001; ANOVA.


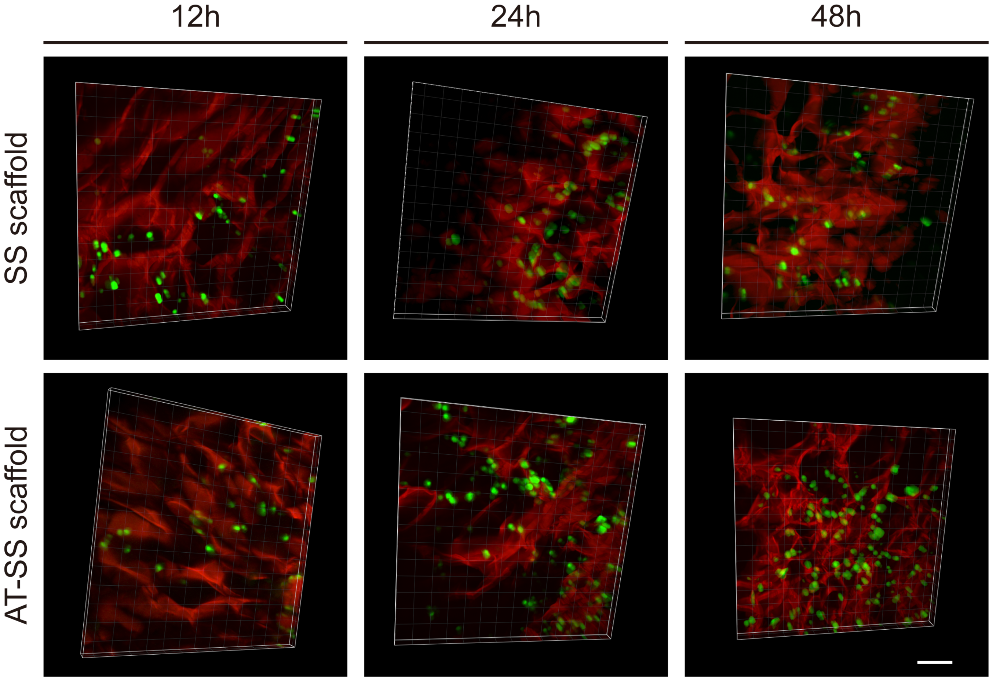


**Figure S10.** GFP-RSC 96 cells were cultured on the SS or AT-SS scaffold for 48 hours. Scale bar, 100 μm.


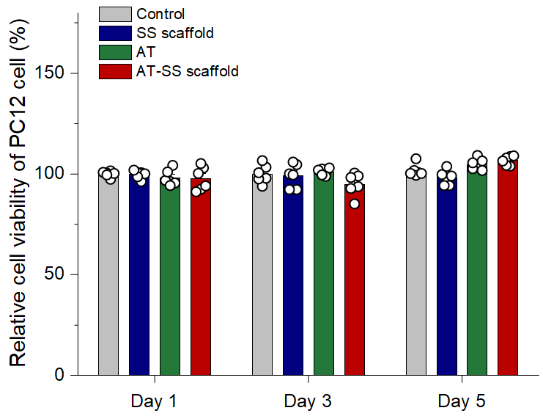


**Figure S11.** The relative cell viability of PC 12 cells after culturing with extracts of AT, SS scaffold, and AT-SS scaffold for 5 days (*n* = 6). Data were presented as mean ± SD.


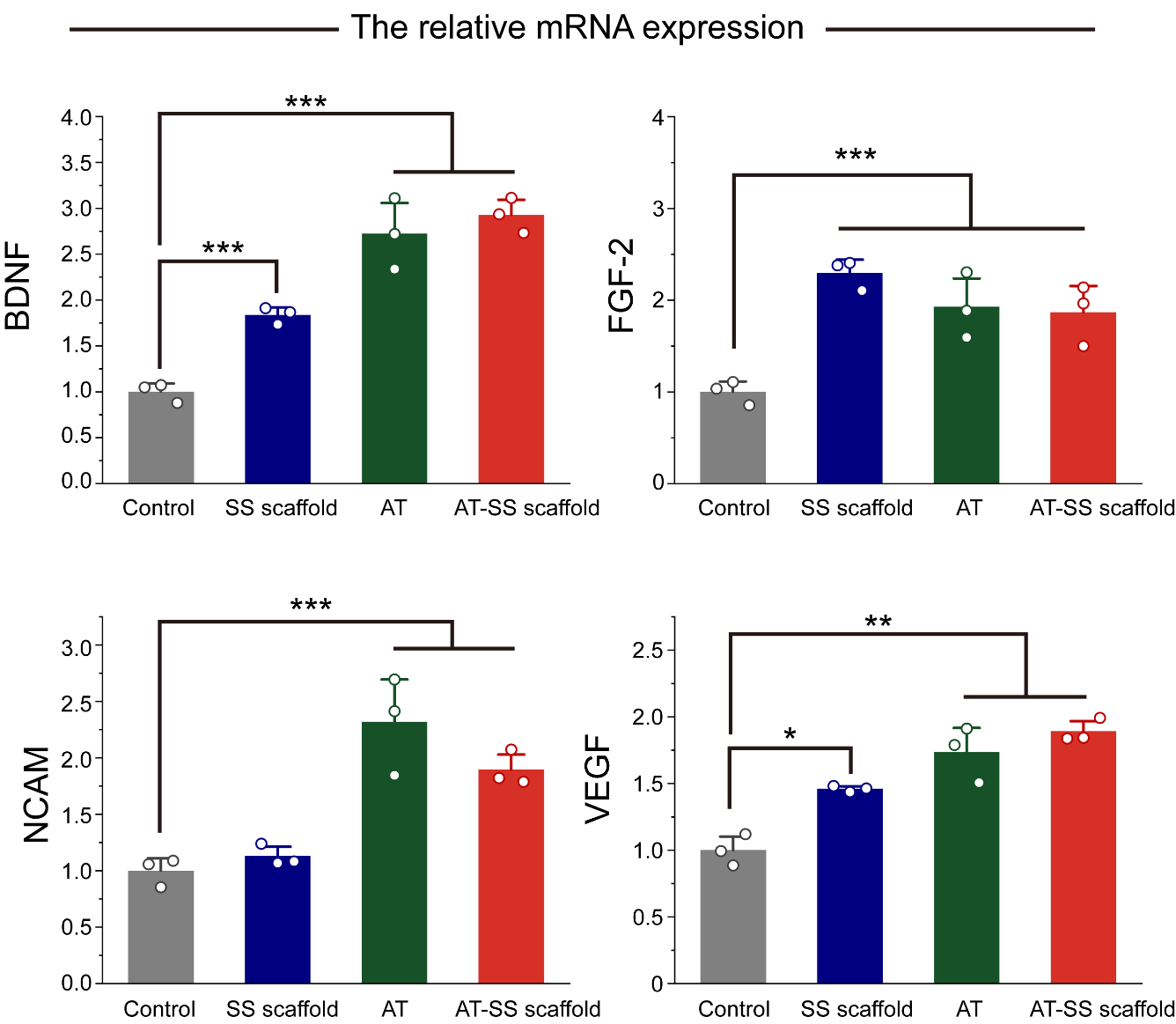


**Figure S12.** The relative BDNF, FGF-2, NCAM, and VEGF mRNA expression of RSC 96 cells cultured with AT-SS scaffold for 24 hours (*n* = 3). Data were presented as mean ± SD; *, *P* < 0.05; **, *P* < 0.01; ***, *P* < 0.001; ANOVA.


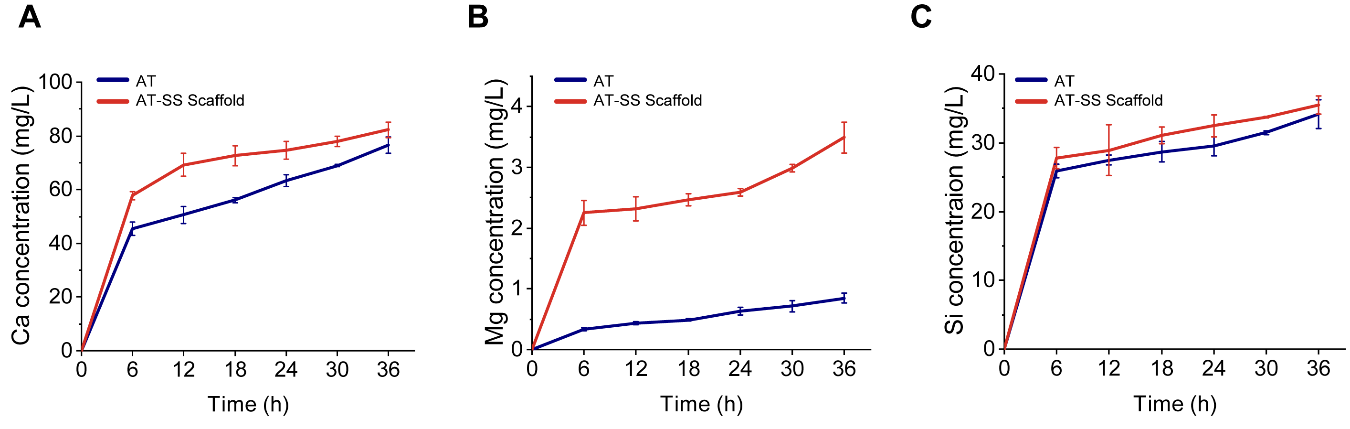


**Figure S13.** The Ca, Mg, and Si ions release kinetics of AT-Scaffold at different time points (*n* = 3). Data were presented as mean ± SD.


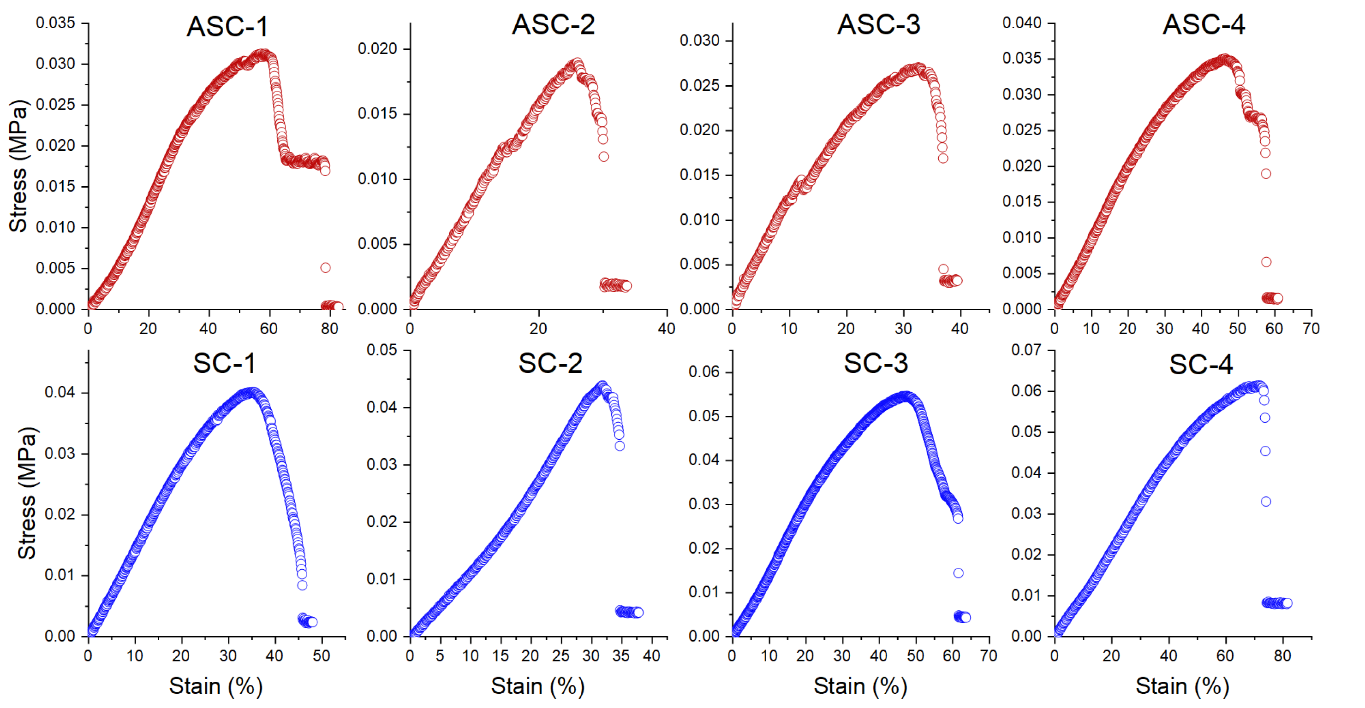


**Figure S14.** The tensile stress-strain curves of ASC and SC.


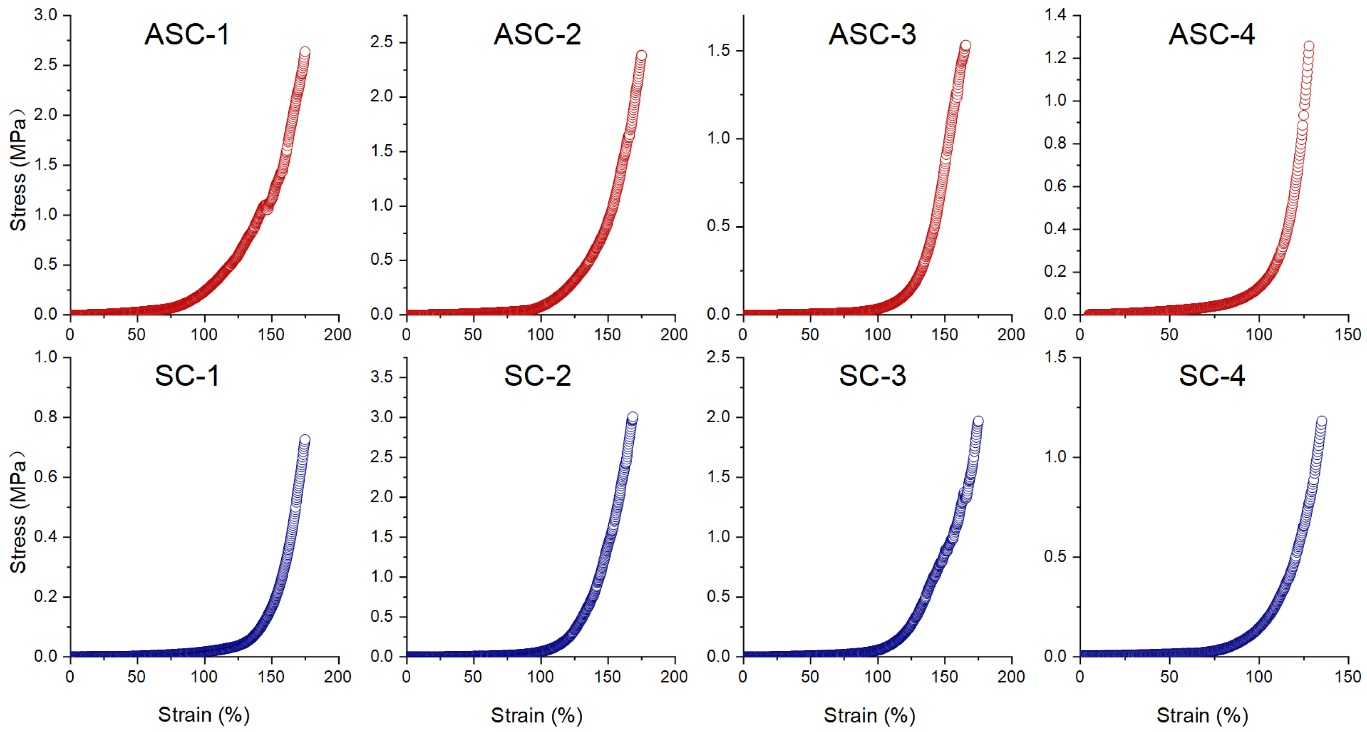


**Figure S15.** The compressive stress-strain curves of ASC and SC.


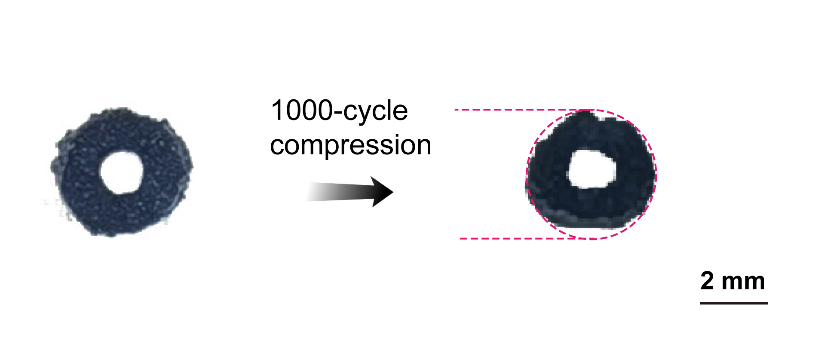


**Figure S16.** The diameter deformation of ASC after 1000-cycle compression. Red dashed lines outline the original external diameter.


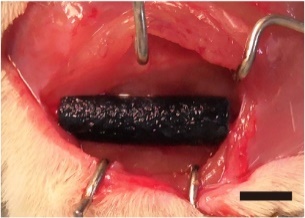


**Figure S17.** Surgery image of conduit transplantation. Scale bar, 5 mm.


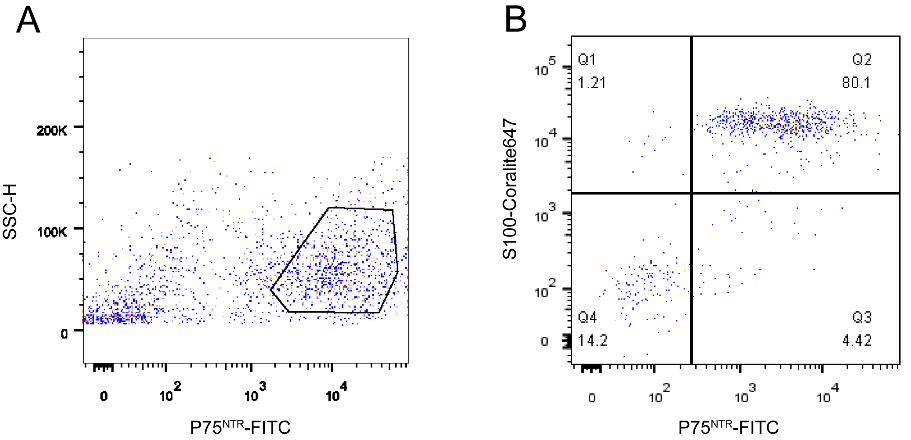


**Figure S18.** Isolation and identification of primary Schwann cells. (A) FACS results of primary Schwann cells stained with P75^NTR^ antibody and FITC-labeled second antibody. (B) Flow cytometry analysis of sorted Schwann cells stained with S100 antibody and Coralite647-labeled second antibody.


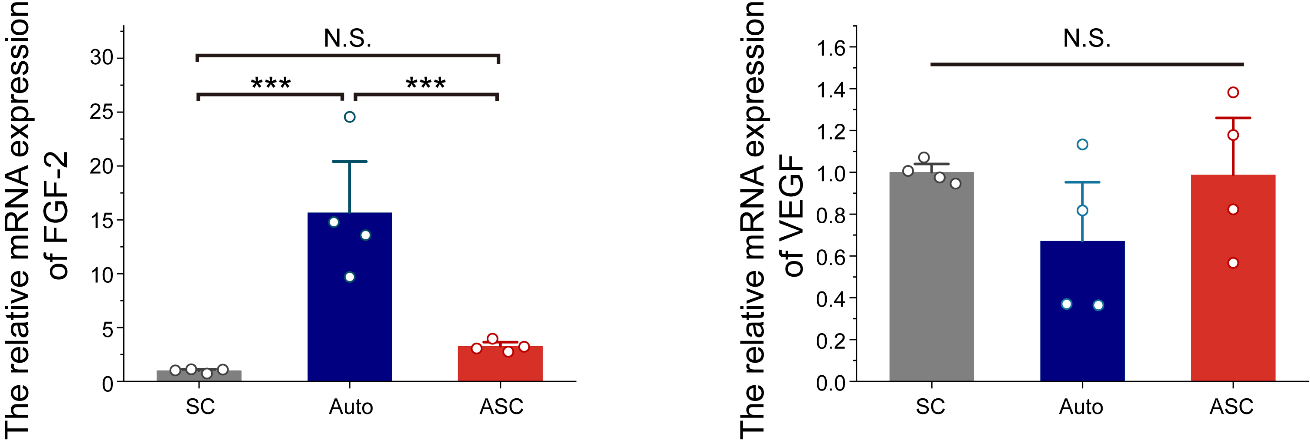


**Figure S19.** The relative FGF-2 and VEGF mRNA expression of primary Schwann cells (*n* = 4). Data were presented as mean ± SD; ***, *P* < 0.001; N.S., not significant; ANOVA.


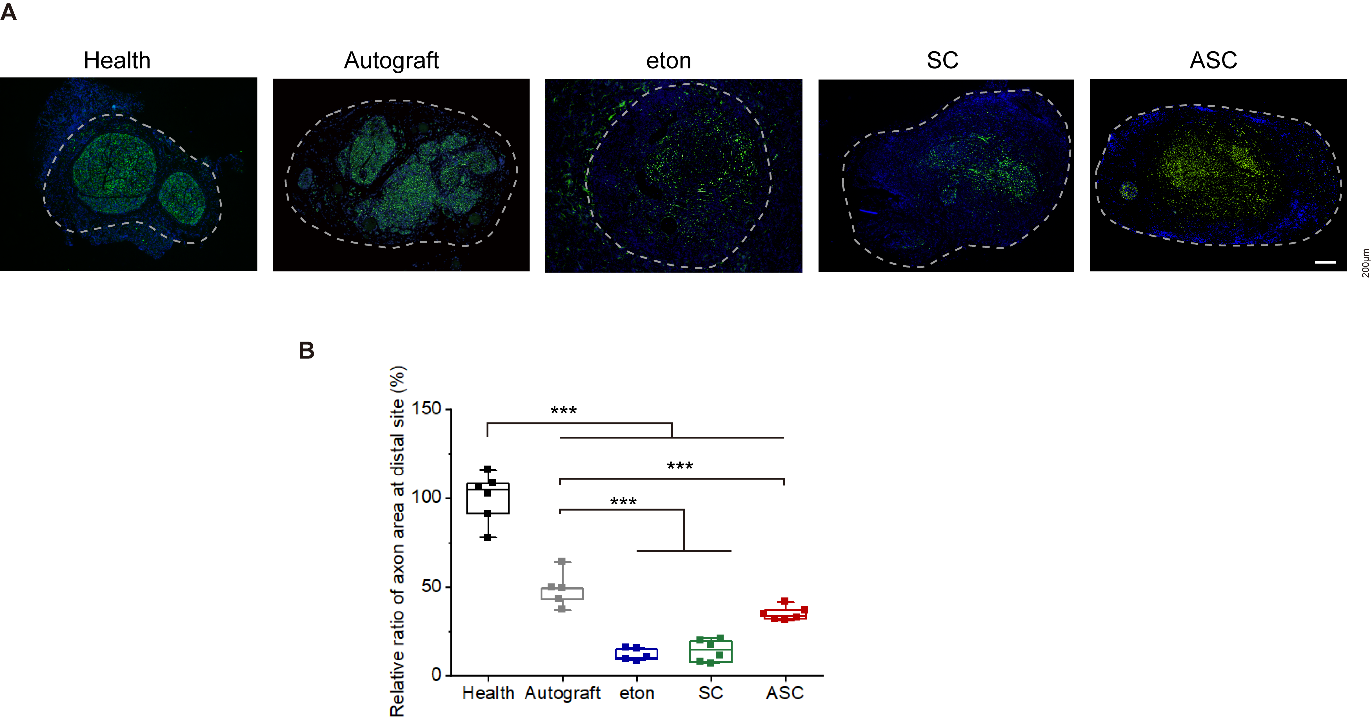


**Figure S20.** Evaluation of axon proportion at distal sites of regenerated nerve 14 weeks after transplantation. Scale bar, 200 μm. (A) Fluorescence images of axons (Tuj-1, green) of transverse sections at the distal site of regenerated and healthy nerves. The regenerated or healthy nerves are outlined with white dotted lines. 6 rats in the health, SC, and ASC groups, and 5 rats in the autograft and eton^®^ groups. (B) The relative ratio of axon area. Data were presented as mean ± SD; ***, *P* < 0.001; ANOVA.


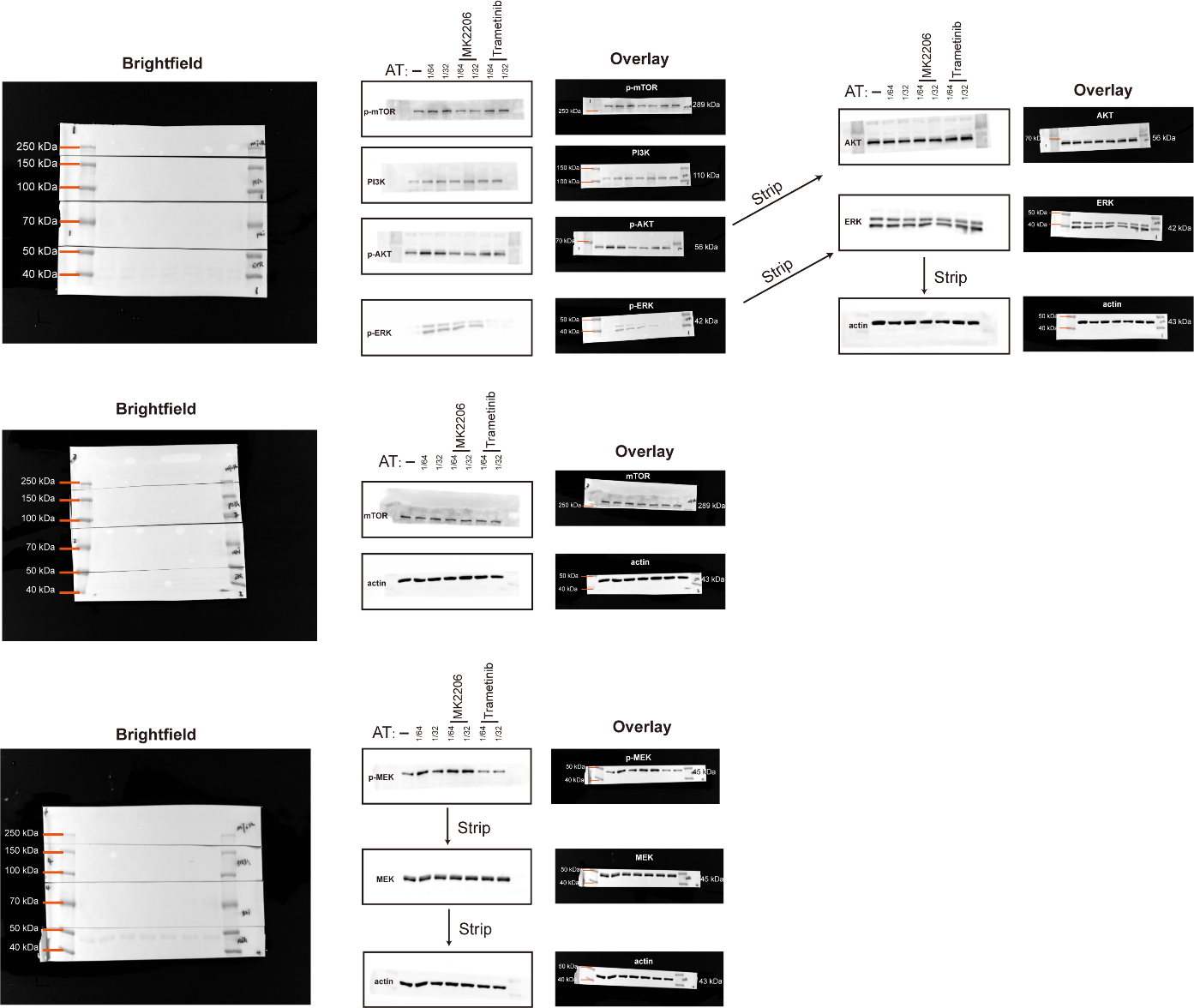


**Figure S21.** Full Western blots associated with Figure 2J in the main text.

**Table S1**. Primers used for the qRT-PCR.

| Gene | Sequence (5’→3’) | |
| --- | --- | --- |
| GDNF | forward | TTATGGGATGTCGTGGCTGTC |
|  | reverse | CGGGCATATTGGAGTCACTGG |
| Integrin | forward | TTGGTCAGCAGCGCATATCT |
|  | reverse | TGGAAAACACCAGCAGTCGT |
| VEGF | forward | CGGGCCTCTGAAACCATGAA |
|  | reverse | GCTTTCTGCTCCCCTTCTGT |
| FGF-2 | forward | GGATCCCAAGCGGCTCTAC |
|  | reverse | AGCTGTAGTTTGACGTGTGGG |
| NGF | forward | ACTCTGAGGTGCATAGCGT |
|  | reverse | TATTGGTTCAGCAGGGGCAC |
| NCAM | forward | GCAGGTAGATATTGTTCCCA |
|  | reverse | GGTTTGGGCTCAGcTTCTCC |
| BDNF | forward | AGCAGAGTCCATTCAGCACC |
|  | reverse | CAGCCTTCATGCAACCGAAG |
| IL-1β | forward | GCAACTGTTCCTGAACTCAACT |
|  | reverse | ATCTTTTGGGGTCCGTCAACT |
| IL-6 | forward | TAGTCCTTCCTACCCCAATTTCC |
|  | reverse | TTGGTCCTTAGCCACTCCTTC |
| TNF-α | forward | GCACAGAAAGCATGATCCGC |
|  | reverse | AACTGATGAGAGGGAGGCCA |
| β-actin | forward | ACAACCTTCTTGCAGCTCCTC |
|  | reverse | CTGACCCATACCCACCATCAC |

GDNF, Glial cell derived neurotrophic factor; VEGF, vascular endothelial growth factor; FGF-2, fibroblast growth factor-2; NGF, nerve growth factor; NCAM, neural cell-adhesion molecule; BDNF, brain-derived neurotrophic factor; IL-1β, interleukin-1β; IL-6, interleukin-6; TNF-α, tumor necrosis factor-α.
